# Hydrogels in Cutaneous Wound Healing: Insights into Characterization, Properties, Formulation and Therapeutic Potential

**DOI:** 10.3390/gels10030188

**Published:** 2024-03-08

**Authors:** Mariana Ribeiro, Marco Simões, Carla Vitorino, Filipa Mascarenhas-Melo

**Affiliations:** 1 Faculty of Pharmacy, University of Coimbra, Pólo das Ciências da Saúde, Azinhaga de Santa Comba, 3000-548 Coimbra, Portugal; marianasmribeiro99@gmail.com; 2 CISUC—Center for Informatics and Systems, University of Coimbra, Pinhal de Marrocos, 3030-290 Coimbra, Portugal; msimoes@dei.uc.pt; 3 Coimbra Chemistry Centre, Institute of Molecular Sciences—IMS, Department of Chemistry, University of Coimbra, 3000-535 Coimbra, Portugal; 4 CIBIT—Coimbra Institute for Biomedical Imaging and Translational Research, University of Coimbra, Pólo das Ciências da Saúde, Azinhaga de Santa Comba, 3000-548 Coimbra, Portugal; 5 Higher School of Health, Polytechnic Institute of Guarda, Rua da Cadeia, 6300-307 Guarda, Portugal; 6 REQUIMTE/LAQV, Department of Pharmaceutical Technology, Faculty of Pharmacy, University of Coimbra, Azinhaga de Santa Comba, 3000-548 Coimbra, Portugal

**Keywords:** hydrogels, wound healing, natural polymers, synthetic polymers, critical attributes, critical analysis

## Abstract

Hydrogels are polymeric materials that possess a set of characteristics meeting various requirements of an ideal wound dressing, making them promising for wound care. These features include, among others, the ability to absorb and retain large amounts of water and the capacity to closely mimic native structures, such as the extracellular matrix, facilitating various cellular processes like proliferation and differentiation. The polymers used in hydrogel formulations exhibit a broad spectrum of properties, allowing them to be classified into two main categories: natural polymers like collagen and chitosan, and synthetic polymers such as polyurethane and polyethylene glycol. This review offers a comprehensive overview and critical analysis of the key polymers that can constitute hydrogels, beginning with a brief contextualization of the polymers. It delves into their function, origin, and chemical structure, highlighting key sources of extraction and obtaining. Additionally, this review encompasses the main intrinsic properties of these polymers and their roles in the wound healing process, accompanied, whenever available, by explanations of the underlying mechanisms of action. It also addresses limitations and describes some studies on the effectiveness of isolated polymers in promoting skin regeneration and wound healing. Subsequently, we briefly discuss some application strategies of hydrogels derived from their intrinsic potential to promote the wound healing process. This can be achieved due to their role in the stimulation of angiogenesis, for example, or through the incorporation of substances like growth factors or drugs, such as antimicrobials, imparting new properties to the hydrogels. In addition to substance incorporation, the potential of hydrogels is also related to their ability to serve as a three-dimensional matrix for cell culture, whether it involves loading cells into the hydrogel or recruiting cells to the wound site, where they proliferate on the scaffold to form new tissue. The latter strategy presupposes the incorporation of biosensors into the hydrogel for real-time monitoring of wound conditions, such as temperature and pH. Future prospects are then ultimately addressed. As far as we are aware, this manuscript represents the first comprehensive approach that brings together and critically analyzes fundamental aspects of both natural and synthetic polymers constituting hydrogels in the context of cutaneous wound healing. It will serve as a foundational point for future studies, aiming to contribute to the development of an effective and environmentally friendly dressing for wounds.

## 1. Introduction

The skin is the largest organ of the human body, accounting for almost 10% of the total body mass [1,2]. It serves as a fundamental anatomical barrier against pathogens and protects the external environment. The skin performs several important functions for maintaining the balance between the biological system and the surrounding environment, such as controlling the thermoregulation process. Furthermore, it is the human organ most frequently injured [1,2,3].

Thousands of years ago, ancient civilizations like the Greeks and Egyptians used tree bark, turmeric, aloe vera, and honey to treat wounds. The increased perception that injured skin is susceptible to contamination and dehydration boosted the development of both synthetic and natural dressings [4].

Since the 1960s, wound dressings have been considered favorable for wound healing because they create an environment conducive to skin regeneration [2]. The application of wound dressings aims to cover the wound, promote re-epithelialization, prevent mechanical trauma, and protect it from infections [5].

The ideal dressing should ensure a moist environment and have the capacity to absorb tissue exudate while allowing gaseous exchange, which is related to its porosity. It must protect the wound against microorganisms and stimulate tissue regeneration. Additionally, it should be rigid enough to allow for fixation on the wound, while remaining flexible and elastic to adapt to body movements. Moreover, it must be biocompatible and biodegradable, ensuring that its by-products are safe. The dressing should provide mechanical stability, and be widely available and cost-effective [1,2,4,5,6,7,8,9].

Due to their intrinsic properties, hydrogels fulfill various requirements for an ideal wound dressing [10]. They offer protection against microorganisms and new lesions [3]. Additionally, they can absorb large amounts of water, up to thousands of times their dry weight [5,11]. Therefore, the highly hydrated three-dimensional (3D) polymeric network allows for the maintenance of a high level of moisture in the wound bed [11]. Moreover, they adhere to the wound but are also easily removable. Their transparency facilitates visual inspection of the wound, and they are customizable and easily adapt to the contours of the wound, promoting autolytic debridement (removal of debris and necrotic tissues), and intrinsically stimulating healing through various mechanisms [5]. These mechanisms include promoting angiogenesis (formation and growth of blood vessels) in wounds with poor perfusion, modulating the immune cells within the wound, or enhancing the migration of keratinocytes and fibroblasts in wound healing [12,13,14,15]. Hydrogels overcome some limitations of traditional treatments, such as prolonged healing, limited body movement, traumatic removal, and poor regeneration of skin attachments [6].

Depending on the type of polymer that constitutes the hydrogel, it can be classified as natural or synthetic. Natural polymers offer better biocompatibility, while synthetic polymers exhibit improved mechanical strength and adjusted properties [3,9]. Regenerative medicine takes advantage of natural polymers, especially as dressings for wound treatment, due to their intrinsic characteristics of biocompatibility and biodegradability. They easily induce tissue repair and skin regeneration because of their interconnected 3D networks embedded in water or biological fluids, as well as their similarity to the extracellular matrix (ECM) [16].

This review provides a comprehensive overview of dressings developed exclusively from hydrogels, which also identifies the critical attributes of an ideal dressing for wound healing. A detailed and in-depth description of various polymers, both natural and synthetic, is also presented, outlining their origins, and alluding to their structure and intrinsic properties. In addition, a critical analysis is provided, which brings together all the critical attributes identified for polymers when used for wound healing. In addition, experimental studies related to wound healing that have used hydrogels in different approaches will be presented, taking advantage of the potential that hydrogels can offer in this context and for different types of applications. This manuscript is a distinctive review that, in addition to presenting detailed information on polymers and hydrogels, also provides a distinct and high-quality discussion about their critical attributes that are key tools for the successful development of wound-healing dressings.

## 2. Wound Healing Process

Wound healing comprises four overlapping phases: hemostasis (blood clotting, stopping bleeding), inflammation (inhibition of microbial growth and wound bed preparation), cellular proliferation (stimulation of fibroblast proliferation and migration to cover the wound surface), and matrix remodeling (strengthening of the tissue and collagen synthesis). These phases cooperate to restore and recover the injured tissue [2,9,17].

A high level of angiogenesis is a critical factor for tissue repair and normal wound healing, as an adequate blood supply ensures the transport of oxygen and nutrients to the wound site [18,19]. Simultaneously, the survival of the new tissue and its integration into the surrounding tissue depends on proper vascularization [20]. It has been observed that fibroblasts cultivated in poly (vinyl alcohol) hydrogels with incorporated arginine, a precursor of nitric oxide, which, in turn, is a key signaling molecule in the regulation of angiogenesis and vasodilation, exhibit increased ECM production compared to fibroblasts cultivated in hydrogels without arginine [4].

The focus of most studies is on the development of absorbent dressings for the treatment of wet or exudative wounds. However, not all wounds require the same care. For instance, burns require continuous hydration and not total moisture absorption [6]. Conventional beliefs advocated for keeping wounds in a dry environment. However, George Winter introduced the concept of moist healing [21]. The wet process accelerates wound healing 3 to 5 times because dehydration (dry process) disrupts the ideal conditions necessary to trigger the wound healing process [1]. The “wet wound healing theory” suggests that a moist healing environment increases the activities of cells and enzymes, facilitates skin cell division, and is advantageous for the formation of granulation tissue, thereby promoting the healing process [21,22]. Therefore, it is crucial to keep the wound environment moist [1,22]. This theory has revolutionized the area of wound management, redirecting research focus from traditional passive drying materials to active moisturizing products [21].

Wound healing can be facilitated by cells, namely fibroblasts, which aid in the formation of the granulation layer, while keratinocytes are involved in re-epithelialization [23,24]. It is well established that fibroblasts and keratinocytes interact synergistically. Previous studies have proven that treatment is more advantageous when applying both types of cells and not just one type. Paracrine signaling provides mechanical, biochemical, and structural properties superior to those of isolated cells. Fibroblasts secrete growth factors that stimulate the growth and differentiation of keratinocytes. Keratinocytes, in turn, secrete molecules that promote the proliferation of fibroblasts [8].

Several growth factors regulate the wound healing process, namely the fibroblast growth factor (FGF), which promotes cell migration and proliferation, as well as angiogenesis; epidermal growth factor (EGF), which improves migration and proliferation of fibroblasts, stimulates epithelization, angiogenesis, and induces the secretion of growth factors by fibroblasts; vascular endothelial growth factor (VEGF), the main growth factor responsible for stimulating angiogenesis; and transforming growth factor (TGF-β), which, in addition to promoting angiogenesis, proliferation, and cell migration, induces the secretion of ECM proteins [25].

## 3. Hydrogel Wound Dressings

The ideal dressing must meet certain requirements to create a conducive environment for wound healing. It should prevent infections, promote the body’s self-healing process, adhere to the wound site without causing damage during dressing changes, and adapt to the body’s movements, among other characteristics. The key properties, which we will refer to as critical quality attributes that an ideal wound dressing should possess, have been outlined in Table 1 [1,2,4,5,6,7,8,9,11].

Hydrogels can play various roles in the field of tissue engineering, such as filling spaces, functioning as wound dressings, or serving as drug delivery systems [8]. Interestingly, the first hydrogel was developed in 1960 by Wichterle and Lim to be used as contact lenses [3].

Hydrogels are promising materials for clinical applications, particularly in the treatment of wounds, due to their intrinsic characteristics that align with the essential aspects an ideal dressing should possess. The primary goal is to stimulate and accelerate healing while enhancing the quality of life for patients [2].

As the prefix “hydro” (water) suggests, hydrogels contain water in their composition, up to 96%. However, the hydrogel sheet itself is not wet. Its hydrophilicity creates a microenvironment in the area of the lesion with an adequate moisture content, which is a critical factor for the rapid healing of the wound. Moreover, moisture is essential to ensure cell viability and proper physiological functioning [2,3,4,8].

The presence of hydrophilic groups (–OH, –SO_3_H, –NH_2_, –COOH, –CONH_2_) is what enables hydrogels to bind to water molecules [3]. The high water content facilitates the transmission of water vapor and oxygen [10,26]. The water vapor transmission rate allows for evaluating the ability of dressings to ensure an adequate level of moisture in the wound. In the case of commercial dressings, it should be between 426 and 2047 g/m^2^/day [27].

In addition to moisturizing tissues, the 3D structure of hydrogels enables them to absorb and retain excess exudate from the wound surface, as well as remove toxins and water-soluble waste from the wound [2,3,10].

The swelling capacity of hydrogels is intriguing because this behavior increases the pore size, facilitating the adhesion and proliferation of cells within the 3D structure. However, this property is only favorable to a certain extent, as it can negatively affect the mechanical properties and integrity of the surrounding tissues [4].

Their dense network per se and their ability to adhere firmly and evenly to the wound surface prevent bacteria from reaching and invading the wound. Hydrogels are very pliable and soft, and their degradation occurs through hydrolysis. They are available in various shapes and sizes, and their flexibility and elasticity allow them to adapt to different parts of the body, making them atraumatic [2,3,10]. In the clinical context, the mechanical stiffness of wound dressings should fall within the range of normal healthy skin, where Young’s modulus varies between 0.42 and 0.85 MPa, allowing for painless movement. If the stiffness falls outside this range, it may compromise the fixation of the dressing to the wound or cause discomfort [6].

Also, the role of the hydrogel depends on the healing phase. For example, during the inflammatory phase, they can intrinsically stimulate the cleaning of the wound bed through the induction of autolytic debridement of the necrotic eschar tissue [2,3,8,10].

Despite the valuable properties of hydrogels and the fact that the polymer network hinders the entry of pathogens, they do not have inherent antimicrobial properties. One of the simplest alternatives is the direct incorporation of an antimicrobial agent, such as minocycline, gentamicin, colistin, or 1% sulfadiazine, either onto the surface or within the hydrogel structure itself (acting as a vector). One problem with these dressings is the rapid release of the drug [3]. In addition to antibacterial drugs, they can effectively transport other bioactive molecules to the wound site. However, the water content decreases as active substances are added [2,3].

Additionally, their structure allows for the deposition and organization of cells, which will differentiate according to specific stimuli and form specific tissue [8]. Moreover, they can release growth factors to promote cell proliferation and stimulate vascular regeneration, aiding in the proliferative phase [5]. Hydrogels also promote the process of keratinization, and their hypoxic and slightly acidic environment promotes angiogenesis at the wound site, inhibits bacterial growth, and attracts cells involved in the wound repair process, such as fibroblasts [2,3]. In addition to participating in the formation of granulation tissue during the healing process, human fibroblasts secrete growth factors, soluble cytokines, and components of the ECM, such as fibronectin, collagen, and hyaluronic acid, that stimulate the proliferation of epithelial cells [8,22].

A study on hydrogels incorporating arginine, a precursor of nitric oxide, which is an important signaling molecule in the regulation of angiogenesis and vasodilation, also demonstrated an increase in ECM production [4,28]. Hydrogels can also be loaded with growth factors. Xiong et al. [29] studied the influence of FGF2 on fibroblast proliferation and found a 40 to 75% increase in the proliferation rate when 100 ng/mL of FGF2 was added. FGF, one of the main promoters of cell proliferation with chemotactic activity, plays an important role in skin healing [4]. Several studies on the incorporation of VEGF into hydrogels have shown improvements in cell proliferation and wound tissue remodeling [30].

Some hydrogel polymers contain RGD (arginine–glycine–aspartic acid) peptide sequences, responsible for interacting with fibronectin and integrin, acting as anchors [6,31]. These sequences are involved in the mechanism of cell adhesion to the ECM and improve cell survival [32], promote migration and proliferation of cells such as keratinocytes and fibroblasts [33], and induce the secretion of growth factors and angiogenic cytokines crucial for ECM remodeling [19]. The type of cell adhesion ligand, particularly RGD peptides, their spatial arrangement within the hydrogel, the combination of several ligands, or the association of ligands and soluble factors can regulate the phenotype and cellular function of the formed tissues [34]. In addition to the described effects, in vitro and in vivo models have demonstrated that RGD peptide enhances the formation of the keratinocyte layer, production of granulation tissue, and strengthening of the wound matrix, thereby improving wound healing [35].

Moreover, their transparency allows for the assessment of even small changes, visual inspection of the wound, and monitoring of the healing process without removing the dressing [2,3,36]. Additionally, removing the dressing negatively interferes with the healing process and should be discouraged [36].

The properties of hydrogel dressings can be enhanced. For instance, the addition of NaCl to the solution during the preparation of type I collagen hydrogels can improve their mechanical properties and optical performance. Similarly, increasing the pH (near the isoelectric point) can enhance their transparency and linear viscoelastic properties [37].

Because hydrogels are designed to come into direct contact with the wound, they should be immunologically neutral [2,3,38]. Hydrogels have shown low rates of adverse effects and low irritation rates [3].

## 4. Polymers

**Figure 1:**
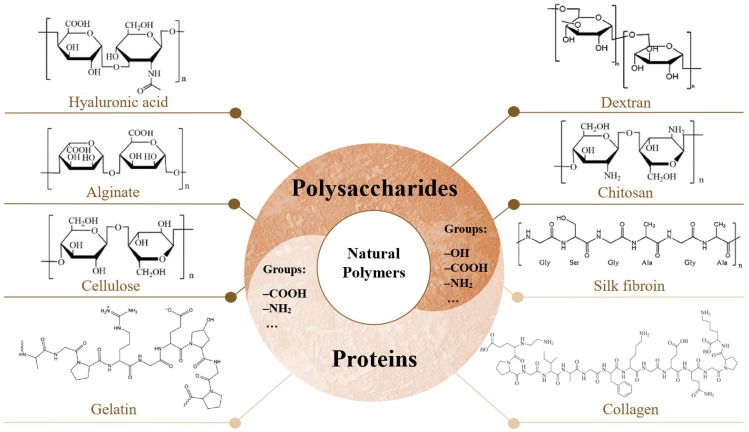
Representation of the chemical structures of some natural polymers classified into proteins (collagen, gelatin, and silk fibroin) and polysaccharides (alginate, hyaluronic acid, cellulose, dextran, and chitosan).

Natural polymers (Figure 1) are biocompatible and often equivalent to macromolecules recognized by the human body [11,17,20]. However, they have relatively low mechanical strength compared to synthetic polymers [11,16,20]. Additionally, they are susceptible to batch-to-batch variations, which may result in slight differences in physicochemical characteristics [11].

**Figure 2:**
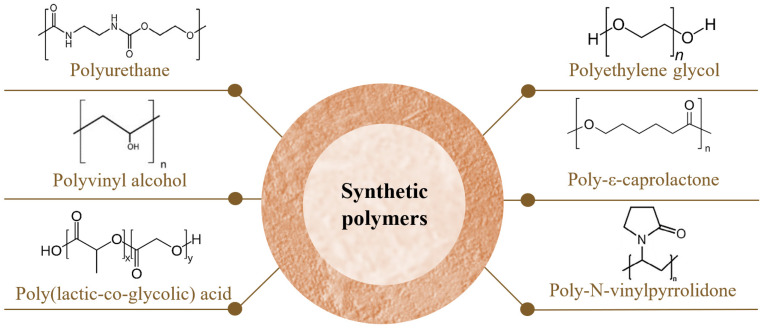
Synthetic polymers and their respective chemical structures.

Synthetic polymers (Figure 2) are chemically synthesized and exhibit controllable and predictable properties. They maintain constant and homogeneous physicochemical properties, often displaying excellent mechanical properties and controlled degradation [5,11,17,39,40]. Some synthetic polymers, such as polyesters, are biodegradable and generally more cost-effective compared to natural polymers [40] and have more abundant sources of raw materials [5]. However, unlike natural polymers, which are biologically inert, synthetic polymers carry the associated toxicity risk and may present biocompatibility issues [17,39,40].

Moreover, these materials often require surface treatment or combination with natural polymers to improve cytocompatibility, as they exhibit weak cellular interactions. Surface treatments aim to reduce hydrophobicity through chemical alterations or enhance cell adhesion by adding adhesion peptides, for example [40]. The most commonly used strategy involves combining synthetic and natural polymers [5,9,40,41], as the latter contribute to their natural biological activity due to their similarity to tissues and the native ECM [20,40]. They mimic the natural microenvironment of cells in the human body, facilitating processes such as cell adhesion, proliferation, migration, and differentiation [20]. Blending polymers improves the mechanical properties of natural polymers. However, their biocompatibility may be somewhat affected [16].

The selection of the polymeric composition of hydrogels should consider the most suitable characteristics according to the type of wound to be treated and the patient’s clinical conditions [2]. 

Table 2 gathers the main sources for obtaining natural polymers and the primary pathways for the chemical synthesis of synthetic polymers.

### 4.1. Natural Polymers

Within natural polymers, we can classify them as proteins (collagen, gelatin, and silk fibroin) and polysaccharides, which can be further subdivided into acidic polysaccharides (alginate and hyaluronic acid), neutral polysaccharides (cellulose and dextran), or basic polysaccharides (chitosan) [16]. Figure 1 schematically represents some of the natural polymers. 

#### 4.1.1. Collagen

Collagen is considered the most abundant protein in the human body [16], constituting approximately 30% of the overall protein content [22]. The main types of collagens present in the body are types I, II, III, IV, and V. This amino acid is ubiquitously present in the ECM [4]. Native collagen is insoluble and needs to undergo a pre-treatment where non-covalent bonds are broken to solubilize and extract it [63].

The main source of commercially used collagen isolation is from marine sources [42], representing an abundant and environmentally friendly reservoir of collagen [64]. The process of obtaining collagen from the transformation of by-products of fish skin and scale is inexpensive and has minimal environmental impact [65].

Although different types of collagen have various structural organizations, they all share the fibrillar triple helix structure [66], composed of the tripeptide sequence (Gly-X-Y)n, where Gly (glycine) accounts for 30% of the total amino acids, and X is typically proline while Y is hydroxyproline [37]. Within the three polypeptide chains, there are repetitive sequences of specific amino acids, such as RGD (arginine–glycine–aspartic acid), which interact with integrins present in cells and promote the adhesion and migration of cells such as keratinocytes and fibroblasts [33,35].

This polymer is highly biocompatible [33,38], eco-friendly [67], and bio-stable. It is a promising candidate for hydrogel applications due to its water absorption capacity, as well as its abundance, and plain structure [4]. They are commonly employed as biomimetic materials for the skin matrix, effectively simulating the natural microenvironment, particularly concerning skin elasticity [68]. Collagen dressings are semi-flexible [69], easily applied and removed, and serve as scaffolds for cytokines and growth factors [4]. Collagen-based hydrogels have demonstrated desirable biodegradability, excellent shape consistency at physiological temperature (37 °C), and good formation of micro and macropores, which are important for cell adhesion and proliferation [20,70]. Collagen-based scaffolds exhibit a high level of hydration and transparency, and provide an appropriate surface area for cellular adhesion, as well as the necessary conditions to ensure their viability, migration, and proliferation [70]. Collagen can polymerize in vitro into a fibrillar hydrogel at physiological temperature, ionic strength, and pH [71]. 

Collagen stimulates the molecular and cellular cascade of wound healing [16], promotes debridement of necrotic tissue [4,16], leads to the synthesis of growth factors that stimulate angiogenesis, and provides a hydrophilic environment that favors re-epithelialization [4]. Collagen acts through a hemostatic effect mechanism [4,46], inducing platelet activation and aggregation, resulting in the release of coagulation factors that lead to the deposition of a fibrin clot at the wound site [33] and increasing the availability of fibronectin, which plays an important role in cell endurance and is essential for the succession of the cell cycle. Collagen plays a crucial role in maintaining cell viability and preserves immune system cells such as macrophages and leukocytes [4].

Collagen dressings inhibit the activity of metalloproteinases (stagnate wound healing in the inflammatory phase), allowing the healing process to proceed [4,46]. In the inflammatory phase, the activation of immune cells stimulates the secretion of pro-inflammatory cytokines, recruiting fibroblasts, endothelial, and epithelial cells. Fibroblasts contribute to collagen synthesis [4,33]. 

At the same time, collagen and its degradation products are responsible for various cellular processes crucial for wound healing, including differentiation, migration, proliferation, and protein synthesis, including collagen itself [35]. Collagen fragments, in turn, release molecules that attract keratinocytes to the wound area [4] and recruit fibroblasts and endothelial cells to regulate granulation and re-epithelialization [4,33,35], aiding in the formation of new tissue [16], which is more resistant to remodeling [35]. Collagen also acts in the remodeling phase (balance between the degradative activity of metalloproteinases and the formation of the new matrix) determining tensile strength [33] and scar formation [33].

Although marine collagen exhibits a high degree of bio-adhesion, biocompatibility, biodegradability, and low immunogenicity [4], it has a lower amount of hydroxyproline amino acids, resulting in lower thermal resistance [72]. When subjected to chemical or enzymatic hydrolysis, it forms marine collagen peptides with low molecular weight, which have higher hydrophilicity, strong calcium affinity, and are more easily absorbed [25]. These peptides possess physiological characteristics that provide activities such as antioxidant and wound-healing properties [25]. Collagen sponges are commonly used in wound treatment. They are highly porous materials and absorb a significant amount of water [4,9]. Their elastic nature and absorbent behavior make them resistant to bacterial infections [4]. However, their rapid degradation limits in vivo use [9].

Collagen-based dressings of avian, bovine, or porcine origin are recommended for the treatment of full- or partial-thickness wounds with minimal to moderate exudate, but they are contraindicated in allergic or sensitive patients and third-degree burns [16].

Collagen-based hydrogel has some limitations, particularly weak mechanical properties [73,74] and tissue adhesion [75]. Furthermore, when in contact with living tissues, it may induce inflammation [74]. On the other hand, the lack of intrinsic antibacterial activity is also a disadvantage, as it alone cannot protect the wound area from infections [76]. In addition to the high cost of pure collagen, which makes it economically unfeasible for large-scale approaches, collagen degradation results in amino acids with the ability to activate the coagulation cascade and with thrombogenic potential, limiting its application as a biomaterial [37]. It can quickly lose its shape and stability due to enzymatic degradation [77]. Moreover, the long gelation time and low mechanical strength of printed collagen-based hydrogels pose additional challenges [78,79].

In a neutral solvent, at a temperature close to physiological, collagen molecules can autonomously assemble into collagen fibers. The hydrogel is then created through the interaction among these collagen fibers [22]. The in vitro degradation of collagen hydrogels occurs by incubating them with a collagenase solution (5 U/mL) at 37 °C, resulting in complete hydrogel disintegration within 3 h [80].

Almeida Cruz et al. [42] compiled the results of several in vivo (animal models) studies on marine-derived collagen. Overall, there was an increase in the amount of collagen in the wound bed, increased granulation tissue, angiogenesis, and promotion of re-epithelialization in the animals with skin wounds, burns, or injuries treated with collagen or collagen peptides extracted from different marine species.

In vitro studies demonstrated that marine collagen peptides isolated from the skin of *Nibea japonica* increased the proliferation and migration of NIH-3T3 fibroblast cells and were classified as non-cytotoxic and hypoallergenic [25,81]. These peptides also showed antioxidant activity against superoxide anion, hydroxyl, DPPH, and ABTS radicals. In vitro studies (scratch wound assay) showed that collagen peptides significantly improved scratch closure rate, and according to another in vitro test (Western blotting), they increased the expression of proteins such as nuclear factor kB (NF-kB) p65, inhibitory-κB kinase (IKK) α, and β, from the NF-kB signaling pathway, which regulates the transcription of genes associated with cellular functions such as adhesion, migration, proliferation, and cell survival. Moreover, they also increased in vitro (Western blotting) the levels of growth factors such as EGF, FGF, VEGF, and TGF-β, all related to wound healing [25]. These results were similar to those obtained with marine collagen hydrolysate regarding skin repair and tissue regeneration capacity. Collagen isolated from *Chum salmon* and *Nile tilapia* skin also demonstrated excellent wound-healing properties [22].

Feng et al. [18] studied aminated collagen hydrogel, isolated from fish scales, in the healing of full-thickness wounds. Aminated collagen is highly biocompatible, minimally immunogenic, eco-friendly, sustainable, and low-cost. The results demonstrated that the hydrogel improved angiogenesis (in vitro) and was effective in wound healing in vivo (rat model). Mature and organized collagen deposition was observed at the wound site, along with re-epithelialization after 14 days, with the formation of tight junctions between the dermis and epidermis, which are crucial for tissue functional recovery.

Previous studies also indicate that hydrogels with higher collagen concentrations are stable, enhance cell viability, and allow for the expression of genes related to matrix macromolecules and cytokines involved in neovascularization and re-epithelialization. This suggests that concentrated collagen hydrogels could be a novel option for cellular therapy in the treatment of chronic skin wounds [82].

#### 4.1.2. Gelatin

Gelatin is a natural polymer resulting from the hydrolysis and controlled denaturation of collagen at high temperatures [6,11,61,83]. It is ubiquitous [84], eco-friendly [67,85,86], sustainable [85], and recyclable [84].

Similar to collagen, gelatin possesses repetitive sequences of amino acids (Gly-X-Y)n, where X and Y are typically proline and hydroxyproline, respectively [17]. Additionally, it contains RGD motifs (arginine–glycine–aspartic acid), which promote cell adhesion, migration, and proliferation. By providing binding sites for integrins, it facilitates keratinocyte migration and improves tissue remodeling [27,83,87,88].

Moreover, the structure of gelatin is flexible and contains numerous free functional groups, such as hydroxyl, amino, and carboxyl groups [31,89], enabling the modification of its structure through chemical conjugation [27].

Gelatin is highly biocompatible and biodegradable [17,27,83,87]. It is hydrophilic, capable of absorbing large quantities of water and exudate [27,90], and mimics the native ECM, making it interesting for wound treatment applications [11,20,38,83,90]. Moreover, its extraction and synthesis are relatively easy [11,91], and dressings based on gelatin exhibit good transparency [91,92,93,94].

Derived from collagen, the main protein of the dermal ECM, gelatin provides the necessary ingredients for dermal regeneration and exerts a positive effect on the biological response [6], without triggering immune responses [6,88]. Compared to collagen, gelatin is cheaper and has lower antigenicity and immunogenicity, as it is partially denatured [27,83]. Gelatin can be used as a drug delivery system or as a scaffold for cell proliferation [17].

This biopolymer positively affects cell viability, mainly due to the RGD peptide sequences, which are responsible for interacting with fibronectin and integrin, acting as anchors and enhancing cell adhesion to the ECM and cell proliferation [6,31]. Despite its cellular adhesion properties, and even though Ma et al.’s study [89] demonstrates that adding gelatin to the polyacrylic acid and polyacrylamide hydrogel enhances the hydrogel’s viscosity and adhesion to surfaces like glass and plastic, the tissue adhesive properties of gelatin are insufficient [90]. Therefore, it is often functionalized with dopamine, imparting adhesive properties to the hydrogel due to its structural resemblance to mussel adhesive proteins [88,95,96]. The key to the good adhesive properties of mussels lies in the abundance of catechol groups [88]. In Wang et al.’s study [96], the addition of 0.06% polydopamine to the gelatin–polyacrylamide hydrogel improved the adhesion of the hydrogels on porcine skin without compromising the ease of removal.

Gelatin undergoes easy and low-temperature processing and exhibits a thermoreversible gelation process [84]. At ambient temperature, gelatin forms hydrogels, but at temperatures above 35 °C, it dissolves in water and forms a transparent gel, due to the loss of hydrogen bonds that connect the chains in a triple helix [17,90]. Furthermore, it has a high degradation rate and is not very viscous above 27 ± 1 °C, which limits its printability. To overcome this low formability, gelatin can be combined with other biopolymers such as alginate and silk fibroin or undergo specific modifications such as methacrylation [11,20]. Gelatin methacrylate, in addition to being a biocompatible and biodegradable polymer, has high thermal sensitivity and facilitates cell migration, making it attractive for wound healing applications. Moreover, its ability for UV radiation-induced photopolymerization provides it with mechanical stability, resulting in high shape fidelity. It also exhibits in situ and rapid gelling [20,87].

Gelatin hydrogels lack antibacterial activity [97], and their stability decreases at high temperatures [98], while they exhibit poor mechanical properties [90,95,97,99]. One solution to improve the mechanical properties of gelatin and reduce its water solubility is through crosslinking. The strength and stability of the crosslinking depend on the crosslinking agent, as well as the water absorption capacity. Recently, crosslinking agents such as lactose, citric acid, and genipin have gained attention due to their biocompatibility. Lactose reacts through the Maillard reaction and results in non-enzymatic glycation of the gelatin chains, while citric acid and genipin react with the amino group, but genipin forms a heterocyclic compound [27]. Lactose has a carbonyl group that reacts with the amino group of gelatin. The resulting structure is more compact, reducing the water absorption capacity. Citric acid has three carboxyl groups. Those that do not react can form hydrogen bonds with polar groups of gelatin. The resulting structure is looser, increasing the water absorption capacity [27].

Ren et al. [100] prepared a biodegradable, recyclable, sustainable, and environmentally friendly gelatin hydrogel, avoiding secondary pollution.

Ionescu et al. [90] prepared a film based on a gelatin derivative containing norbornene functionalities and evaluated its wound healing potential in vivo (rat model). The results showed that there was no significant degradation during the 3-week study period. There was a considerable improvement in the wound healing process. Additionally, a high percentage of wound contraction (80% ± 0.3) was observed at the end of 12 days.

#### 4.1.3. Silk Fibroin

Silk fibroin (SF) is a natural protein-based biopolymer [54,60,101,102,103,104,105,106,107,108,109,110,111,112]. Silk fibers are primarily composed of proteins derived from silkworms, namely fibroin, sericin, and 18 different amino acids [56,113]. Among these, alanine, glycine, and serine residues make up the majority of the amino acid sequence [54,56,101].

Chemically, this fibrous bioproduct possesses abundant amino groups (primary amines), hydroxyl, and carboxyl groups along its molecular chain that are accessible for chemical modifications [101,107,112,114].

Furthermore, this structured amphiphilic copolymer is composed of alternatively repeating units of hydrophilic light chains and disulfide-connected hydrophobic heavy chains [60,101,115]. In solution, SF adopts metastable amorphous conformations such as random coil and α-helix structure (Silk I). When exposed to physical and chemical stimuli, such as shear forces, high silk concentration, low pH, high temperature, vortexing, sonication, cross-linking agents, blending with other polymers, or organic solvent treatment, the amorphous portion of SF can be transformed into stable and insoluble anti-parallel crystallized β-sheets (Silk II) [106,114,115,116,117,118].

In physiological conditions, the SF protein adopts a low-energy β-sheet conformation and tends to aggregate, resulting in hydrogel formation through self-aggregation. This self-assembly approach is relatively simple. However, it can be time-consuming [104,110]. Another technique used to induce hydrogel formation is the ultrasonication technique. The formation of β-sheets occurs through alterations in hydrophobic hydration. The application of ultrasound waves promotes intermolecular interactions of fibers, leading to a structural rearrangement of the protein. This process involves conformational changes from a random coil to a β-sheet, culminating in hydrogel formation [104,110,115]. Previous studies have shown that this technique reduced the gelation time of SF from days to minutes, with the formation of hydrogels reported within 15 to 45 s [110,115].

In general, the SF hydrogel state is preferred for its adaptability and ease of use [111]. The conformation of the SF chain determines the morphological structure and solubility of the hydrogel. The higher the concentration of SF, the greater the content of the β-sheets (Silk II) [117]. In turn, the crystalline Silk II structure imparts greater mechanical resistance and long-term stability to the hydrogel [104,113,116]. The hydrophilic blocks enhance toughness and elasticity [113].

SF possesses excellent biological properties, including outstanding biocompatibility both in vitro and in vivo, and tunable biodegradability, forming non-inflammatory by-products. It also exhibits high tensile strength and robust mechanical properties, along with excellent flexibility, elasticity, and malleability [16,17,54,57,60,104,105,106,107,108,110,112,113,114,116,117,119,120]. Moreover, it is naturally abundant, bio-sustainable [121], and eco-friendly [121,122,123].

In addition to addressing biodegradation issues associated with non-biological materials [115], SF can act as a natural strengthening agent, providing the desired mechanical strength and hardness, instead of relying on synthetic polymers and chemicals [106,110,113]. Studies have shown that the addition of small amounts of SF improved the physical and mechanical properties of a collagen scaffold without affecting its biological nature [106].

The SF hydrogel has become a promising biomimetic dressing, given its similarity to Young’s modulus of the skin and its ability to adjust mechanical properties to match the desired resilience and elasticity of native tissues. In addition to supporting cell proliferation, it allows for the controlled release of antibacterial molecules and bioactive compounds for skin wound regeneration [110,112,114,117,120].

Furthermore, its low immunogenicity, non-toxicity, good accessibility, low cost (especially when compared with collagen due to the rigorous processing associated with collagen extraction), easy processability, outstanding stability, and chemical modifiability significantly contribute to its application in the biomedical field [54,57,102,108,110,112,115,116,117,120,124,125].

Moreover, due to the presence of hydrophilic groups on SF backbones that are easily hydrated, such as hydroxyl and carboxyl groups capable of forming hydrogen bonds with water molecules [101,112,114], SF hydrogels can absorb exudate and maintain a moist environment. This helps keep the wound area hydrated, promoting cell adhesion and migration [16,17,107,109,112,114,116].

SF hydrogel dressings exhibit excellent inherent potential in wound healing [17,60,102,107,110,112,116,117,118,124]. They demonstrate a good water vapor transmission rate, water retention capacity, gelation behavior, and proper oxygen permeation. Additionally, SF hydrogel dressings possess hemostatic properties and support the recruitment of various cell types, including endothelial cells, macrophages, neutrophils, fibroblasts, and keratinocytes. Furthermore, they promote cell proliferation and migration, facilitating re-epithelialization and the formation of granulation tissue [17,104,105,106,108,109,112,119,126].

In addition to numerous studies reporting the effectiveness of SF hydrogels in drug delivery and the regulation of growth factors [60,104], the literature documents indicate that SF can induce the release of EGF [107].

SF has excellent cell adhesion [125]. In vitro studies have demonstrated that SF-based hydrogels provide mechanical support and act as a matrix for tissue formation, significantly enhancing cell adhesion and proliferation. This effect may be attributed to their capacity to provide nutrients, promote cell–cell interactions, and facilitate the spreading of normal human keratinocytes and fibroblasts under in vitro conditions [17,54,101,107,109,115].

Moreover, SF-based scaffolds downregulate the expression of pro-inflammatory cytokines, suppressing inflammation [115]. Low-molecular-weight SF can activate the intrinsic coagulation cascade, acting during the hemostasis phase [56].

Furthermore, previous studies have shown that SF is used due to its capacity to stimulate collagen synthesis and contribute to the production and deposition of ECM components. This makes a direct contribution to the wound repair and tissue regeneration process [101,107,108,110,112,119].

NF-kB is an important mediator of inflammatory responses. The NF-kB pathway is crucial in the activation and differentiation of innate immune cells and inflammatory T cells [102]. SF can activate the NF-kB signaling pathway through the expression of cyclin D1, vimentin, fibronectin, and VEGF. In addition to modulating cellular activities such as cell adhesion, differentiation, and proliferation, this activation promotes ECM deposition and regulates inflammation and ROS elimination. This signaling pathway is closely related to wound healing and constitutes the underlying mechanism in the healing process of SF-based dressings [102,104,110,116].

Despite the highlighted promising inherent properties, SF hydrogels present some limitations, such as the lack of angiogenic activity [115], and antibacterial and antioxidant properties [110,115,116,124]. Additionally, they have a high gelation time [98].

As a single component, SF may not be sufficient for skin regeneration due to the lack of cell-specific binding sites [109]. Furthermore, the high cutaneous affinity of SF hydrogels allows them to adhere easily to the skin surface without the need for biological adhesives [105,117]. However, in the case of wounds with irregular contours, additional auxiliary fixation is required since they cannot adapt to deformation [111].

The transparency of SF hydrogels sparks controversy. Some authors acknowledge their good transparency [121,125], while others assert that the production of hydrogels leads to microstructures lacking in transparency [127].

The degradation of silk protein is carried out through proteases present in the biological system. The degradability and degradation rate depend on the type of silk, the concentration of SF, and the content of β-sheets [106].

Guan et al. [118] developed an SF-based hydrogel and investigated its mechanism for skin repair and wound healing in a second-degree burn mouse model. The results showed that the SF hydrogel provided a moist environment conducive to wound healing. It improved adhesion and migration of fibroblast and keratinocyte cells in vitro through the Talin 1 pathway, which is closely related to the healing process, and associated proteins (vinculin, paxillin, p-FAK, and FAK). The interconnected porous morphology of the hydrogel, suitable for cell growth, facilitated nutrient delivery to cells and promoted the absorption of cellular debris or metabolic waste. In the in vivo study, the treated wound appeared smooth and moist, with no apparent inflammatory reactions. After 12 days, the burn skin treated with SF hydrogel exhibited morphology and histology similar to normal skin, showing complete wound regeneration without edema or granulation tissue. The hydrogel-treated group demonstrated faster re-epithelialization and wound closure compared to the control groups.

Li et al. [117] investigated the therapeutic effect of an SF-based hydrogel on hypertrophic scars in vivo using rabbit ears. Hypertrophic scars, occurring in 33% to 91% of burn victims, lack an established optimal treatment method. The findings of the study suggest that the SF hydrogel exhibits outstanding inhibitory effects on hypertrophic scars, reducing their thickness and lightening their color. These effects were attributed by the authors to the downregulation of α-smooth muscle actin (α-SMA) expression levels. Additionally, the hydrogel-treated group demonstrated lower collagen fiber density and more organized arrangements than the blank control and scar removal cream groups.

#### 4.1.4. Alginate

Alginate is a natural polysaccharide primarily found in the cell walls of marine algae (macroalgae) in the form of alginic acid. Additionally, it can be obtained from bacteria [34,44]. Bacterial biosynthesis enables the production of alginates with more defined physical properties and chemical structures compared to those derived from marine algae. Moreover, the ability to manipulate bacteria has allowed for the customization of alginate characteristics [34].

Alginate is an unbranched linear anionic polymer composed of repeating units of (1,4)-linked α-L-guluronic acid and β-D-mannuronic acid, referred to as G blocks and M blocks, respectively. Depending on the source from which alginate is extracted, these two types of monomeric acids may be present in varying proportions and assume diverse structural arrangements, thereby influencing the properties of alginate [1,21,34,44].

It is believed that only the G blocks are involved in the intermolecular binding with di and trivalent cations [34,44]. A higher content of G blocks enables the formation of stiffer hydrogels with improved mechanical properties [21] due to the cationic interaction they establish with ions such as calcium, forming a structure resembling an “egg-box” [1,38]. On the other hand, a hydrogel with a higher content of M blocks tends to be less adhesive and is capable of stimulating human monocytes to produce cytokines, making it applicable in wound healing processes [1].

Alginate is an insoluble salt, but it can be extracted through treatment with aqueous alkaline solutions, typically NaOH, resulting in the formation of water-soluble sodium alginate [1,34].

It is a highly porous, hydrophilic, moisture-permeable, biostable, highly biocompatible, biodegradable, and non-toxic polymer. Furthermore, it is widely recognized as safe, readily available in nature, inherently non-adhesive, easy to process, and possesses good resistance in acidic media [1,21,38,39,44,53,128,129] and transparency [78,92]. Moreover, alginate is renewable [130,131], has an eco-friendly nature [131,132,133,134], and is cost-effective [1,39,44,129,130,132,134]. However, it still has some shortcomings, including weak mechanical properties and cell adhesion [1,21,44,128], the inability to promote angiogenesis, inhibit microbial infections, slow hemostasis, and difficulty in reducing scar formation [129].

While some researchers have found little to no immune response, others have reported that alginates with a high content of M blocks were immunogenic. This observed immunogenic response was attributed to residual impurities in the alginate itself, such as endotoxins, polyphenolic compounds, and heavy metals, due to its natural origin [34]. Orive et al. [135] demonstrated that purified alginates had fewer impurities and did not induce any significant reaction when implanted in animals (mice).

Alginate is widely used either in its hydrated gel form to provide moisture to dry wounds or in its dry form for wound fluid absorption [34]. It is highly absorbent, particularly in a lyophilized form, capable of absorbing water or body fluids up to twenty times its weight [16,21]. It is especially useful in wounds with moderate to high exudate levels [1,44,77]. Alginate can channel wound exudate towards the surrounding healthy skin, a phenomenon known as “lateral capillarity”. However, research indicates that a high content of M blocks hinders this effect [21].

Calcium alginate-based biomaterials (insoluble in water) tend to partially dissolve when in contact with body fluids. As they absorb wound exudate, an ion exchange occurs between calcium (from the alginate) and sodium (from body fluids), releasing calcium ions. This results in the formation of a solubilized gel (sodium alginate), which is conducive to wound healing, providing a moist microenvironment in the wound area that stimulates re-epithelialization, assists in granulation tissue formation and minimizes bacterial infections. They promote autolytic debridement and do not adhere to tissues, making their removal almost painless, accelerating wound closure, and preventing secondary injuries during dressing changes [1,6,16,21,34,38,53,128]. However, the ion exchanges that occur under physiological conditions limit the long-term stability of ionically crosslinked hydrogels [34].

Alginate biopolymers can be applied as drug delivery systems [1,21,34] and are commonly used in biomedical products for the treatment of bleeding/exudating skin wounds and burns due to their hemostatic properties [16,53,77]. On one hand, calcium ions released under physiological conditions promote hemostasis, and on the other hand, the hydrogel itself allows for the aggregation of platelets and red blood cells within its matrix [34]. Alginate stimulates macrophage activation and induces the production of interleukin-6 and tumor necrosis factor α by monocytes, initiating the second phase of the wound healing process, the inflammatory phase [21,128].

The removal of alginate-based dressings causes less pain than traditional dressings and will not induce additional harm to the wound site [1,21]. However, its weak adhesive properties require a secondary dressing to protect and attach it to the wound area [16,53]. Since the inherent cell adhesion properties of alginate are deficient [20,98], recently, alginate derivatives have been prepared by chemically introducing adhesive cell peptides, including the RGD sequence and others like YIGSR (Tyr-Ile-Gly-Ser-Arg) and DGEA (Asp-Gly-Glu-Ala) [34].

Alginate exhibits relevant rheological properties such as viscosity, rapid gelation, and the ability to stabilize dispersions [1,98]. However, it has weak mechanical properties and low tensile strength, which limits its application in wound healing [98,128]. Bahadoran et al. [136] prepared a sodium alginate/polyvinyl alcohol hydrogel and observed that an increase in alginate concentration resulted in more porous structures with enhanced swelling capacity, improved elasticity, and degradation rate.

Increasing the molecular weight is one way to enhance the physical properties of the resulting hydrogel. However, an increase in molecular weight raises the viscosity of the alginate solution, which may compromise the viability of cells or proteins incorporated into the mixture due to generated shear forces. The viscosity of the pre-gelation solution and post-gelation stiffness can be controlled independently, so a combination of low- and high-molecular-weight polymers can be used to increase hydrogel elasticity with a minimal increase in solution viscosity [34]. The viscosity of alginate solutions remains unchanged within pH values between 5 and 11. When the pH falls below 5, protonation of the –COO^−^ groups in alginate occurs, reducing electrostatic repulsion between alginate chains, facilitating their approximation, and forming hydrogen bonds, leading to increased viscosity. When the pH exceeds 11, depolymerization occurs, decreasing viscosity [34].

The functional groups of alginate enable it to easily cross-link with other biopolymers, forming a network that enhances the physical stability of the dressing. The ionic nature of alginate promotes the formation of bioactive interpolymeric complexes with compounds such as chitosan, a cationic polysaccharide [1].

#### 4.1.5. Hyaluronic Acid

Hyaluronic acid (HA) is an animal-derived glycosaminoglycan and is a structural component abundantly present in the ECM found in embryonic mesenchymal tissues [7]. This linear polyanionic heteropolysaccharide consists of alternating units of β-1,3-N-acetyl-D-glucosamine and α-1,4-D-glucuronic acid [17,137].

HA is a highly biocompatible, biodegradable, non-toxic polymer that can be easily chemically modified [7,9,19,38,137,138]. It is a non-adhesive, non-immunogenic [139], and eco-friendly glycosaminoglycan [140,141] with high transparency [142,143,144]. In the architecture of the ECM, HA plays regulatory roles in water homeostasis, ensuring proper tissue hydration during wound repair [137,138]. It is a semi-flexible [145] and water-soluble macromolecule. HA is one of the most hygroscopic molecules, capable of absorbing and retaining large amounts of water, increasing its volume by up to 1000 times [7,142]. It has several carboxyl and hydroxyl groups in its structure, which impart a highly hydrophilic character to it, enabling it to absorb exudate [45]. It provides a moist environment and allows for oxygen permeation [142,146]. At physiological pH, HA carries a negative charge. The acetamido and carboxyl groups in its structure form hydrogen bonds with water molecules, stabilizing the polymer’s secondary structure [45].

Most approaches to obtaining HA hydrogel dressings involve high costs and tedious multi-step reactions [45,146]. Recently, advances in HA extraction technologies have led to a more stable and cost-effective form of HA [142].

HA-based hydrogels are reabsorbable and easy to scale up [98]. However, HA hydrogels have relatively weak mechanical properties [45,147], require crosslinking for stability [98], exhibit low adhesion strength in humid environments [147], and undergo rapid enzymatic degradation (through the action of hyaluronidases) in physiological environments [39,45]. However, its esterification enhances its stability, mechanical properties, and degradation rate [7,45]. Moreover, crosslinking gelatin with HA-based products allows for increasing the initial HA degradation time from one week to several months [31]. Clinical cases will be presented for two examples of medical devices that use ester derivatives of HA later on, namely, Hyalomatrix and Hyalosafe. This chemical modification reduces hydrophilic components and increases hydrophobic groups, aiming to enhance its stability while maintaining its biological properties and initial safety profile. Solubility depends on the percentage of esterification, which is a controllable process. In vivo data have demonstrated that the degradation of this material is safe and occurs through ester bond hydrolysis, resulting in gradual polymer hydration, making it increasingly resemble native HA. Furthermore, studies have confirmed its biocompatibility. Hyalomatrix and Hyalosafe are examples of two medical devices that use ester derivatives of HA. When used in burns, these are alternatives that promote functional recovery and effective repair of the burned area [7].

HA accelerates wound healing through various mechanisms [137]. It provides 3D support to the extracellular space, is involved in the proliferation and migration of cells such as fibroblasts and keratinocytes, stimulates angiogenesis, enables important complex interactions for the healing process, and contributes to the organized and structured construction of newly formed tissue. It is a valuable option, especially for application in deep burns, due to its properties that facilitate nerve regeneration while simultaneously limiting scar formation [7,19].

High concentrations of HA have been reported in processes of regeneration, remodeling, and morphogenesis. Interestingly, this molecule is involved in the early stages of wound healing and tissue repair, assisting in the organization of endothelial cells and fibroblasts at the site of injury [7].

In addition to its role in organizing the ECM, depending on the molecular weight, HA can perform different biological functions [7]. High-molecular-weight HA allows for the formation of structures with increased stability, viscosity, and viscoelasticity. It is considered low-molecular-weight if values are below 1–25 × 10^4^ Da, and high-molecular-weight if values are above 1 × 10^6^ Da [45].

After injury, platelets release high-molecular-weight HA, which accumulates at the wound site and leads to fibrinogen deposition and clot formation. HA recruits neutrophils involved in the removal of dead tissue and phagocytosis of debris and induces the release of interleukin 1β, interleukin 8, and tumor necrosis factor-α. It modulates the inflammatory phase of the wound healing process, exerting an anti-inflammatory effect and regulating early inflammation [45,137]. Experimental models have demonstrated that high-molecular-weight HA inhibits angiogenesis, preventing the supply of oxygen and nutrients, and consequently, tissue regeneration [7,148]. The secretion of inflammatory cytokines contributes to the fragmentation of high-molecular-weight HA into low-molecular-weight HA, which, in turn, recruits monocytes and leukocytes. It modulates inflammation by its activity on free radicals, its antioxidant effect, and the exclusion of lytic enzymes from the cell. Low-molecular-weight HA is pro-angiogenic, stimulates the production of pro-inflammatory cytokines and growth factors responsible for ECM remodeling, and regulates the migration and proliferation of fibroblasts and keratinocytes, aiding in the proliferative and remodeling phases of the wound healing process. Fibronectin and low-molecular-weight HA play an important role in wound contraction, as they induce their differentiation into myofibroblasts and guide fibroblast proliferation, essential for collagen deposition, which will form the new granulation tissue matrix, rich in HA itself. In the re-epithelialization phase, low-molecular-weight HA interacts with keratinocytes, regulating the re-epithelialization process [7,45,137]. Furthermore, previous studies have demonstrated that enzymes involved in the degradation of HA promote cell proliferation, providing additional evidence that HA must be broken down to enhance cell growth [148].

Some studies suggest that HA is not suitable for cell adhesion and proliferation [45,145]. While some authors attribute the inability to support cell attachment to insufficient strength [145], others presume that it is due to the thermodynamic polyanionic and hydrophilic characteristics of HA materials, hindering the adhesion of cells to anionic surfaces. Biomaterials based on high-molecular-weight HA have shown lower adhesion compared to lower-molecular-weight counterparts and are, therefore, used in situations where preventing adhesions is desired, such as post-surgery [31]. Cross-linking it with biopolymers containing free amine groups, such as gelatin, can enhance its cell adhesion and proliferation properties due to the much more stable amide bond compared to ester bonds [31]. The main agents for cross-linking HA chains include carbodiimides, polyfunctional epoxides, hydrazides, sulfides, and aldehydes. Carbodiimides (water-soluble) are the most commonly used due to their non-cytotoxicity, biocompatibility, and the fact that they are not incorporated into the structure after cross-linking. Additionally, their cross-linking reaction is gentle and easily controlled [31].

The rheological properties of HA depend on the pH, temperature, and ionic strength of the solution. HA undergoes hydrolytic degradation when the pH of the solution is below 4 or above 11, resulting in a decrease in viscosity and the integrity of the polymer network. This aspect is significant because the pH of the wound varies during the healing stages. After an injury, the pH increases at the wound site, reaching approximately eight and gradually decreasing as the healing process progresses until it reaches five when healing is complete [45].

Based on current clinical experience, medical devices incorporating HA are considered a safe and effective therapeutic alternative in burn treatment, demonstrating superior outcomes in wound healing compared to standard care [7].

In the context of wound healing, exogenous HA has been the subject of investigation, with promising results emerging. Preliminary in vivo studies have shown that the topical application of HA promotes skin regeneration in hamsters and rats. However, this polymer possesses limited properties regarding its residence time and solubility, necessitating chemical modifications to enhance resistance to degradation, prolong the in vivo residence period, achieve other physicochemical characteristics, and explore different production methods [7].

A retrospective study involving 11 burn treatment centers was conducted, encompassing 57 patients, with 31 presenting deep partial-thickness burns and 22 full-thickness burns. Hyalomatrix was applied, and medical follow-up was extended for 37 days. Notably, after 7 days, enhanced re-epithelialization was observed in cases of deep partial-thickness burns. By the 37th day, complete wound closure had been achieved in 85.7% of patients. Hyalomatrix, an advanced, flexible, and conformable dressing, consists of two layers: a thin, transparent silicone layer on top and a 3D fibrous matrix layer in contact with the wound, composed of ester-derived HA. Upon contact with the wound, the biodegradable fibrous matrix spontaneously integrates and undergoes hydrolysis, releasing HA [7]. In a comparable study involving 300 individuals with deep partial-thickness burns treated using Hyalomatrix, burns healed within 21 days for 83% of cases. Hypertrophic scars, evident in almost all individuals, disappeared within one year post-healing in 90% of patients and after two years in 96% of cases. The incidence of infections decreased from 29.5% to 10% [7]. The study’s findings underscore the effectiveness of Hyalomatrix as a regenerative matrix, providing support for cell migration and deposition from the wound margins while promoting the organization of constituents within the matrix, including fibroblasts and endothelium in the injured area. The top layer serves as a physical barrier against microorganisms, prevents excessive fluid loss, and allows for the monitoring of the healing progress without necessitating dressing removal due to its transparent nature. Importantly, this layer was designed not to cause pain or damage to newly formed tissue upon removal, demonstrating a favorable safety profile, even in pediatric populations. For burn victims undergoing surgical wound preparation involving necrotic tissue removal, this advanced device safeguards the residual dermal layer and stimulates tissue regeneration from the wound margins and cutaneous appendages [7].

Hyalosafe, another HA ester, serves as a dressing specifically designed for effective coverage in treating first and superficial second-degree burns. This transparent HA film is directly applied to the wound, creating and maintaining an optimal level of moisture in the wound area. This, in turn, establishes favorable conditions for rapid epithelial renewal without the risk of tissue maceration. The degradation of ester bonds releases HA, actively promoting re-epithelialization. Importantly, this membrane is non-adherent, ensuring a painless removal process [7].

Facial burns pose significant challenges, often requiring hospitalization and accounting for 25% of pediatric burns. Due to the unique characteristics of facial tissue, there is a notable risk of fluid loss. A study involving 40 children demonstrated that Hyalosafe exhibits excellent wound-healing properties in second-degree facial burns, leading to significant aesthetic outcomes. Remarkably, there were no reports of wound infection [7].

#### 4.1.6. Cellulose

Cellulose stands as the most abundant biopolymer obtained from natural sources [47,51,52,149,150]. It is considered the safest material on earth and an endless reservoir of resources to develop environmentally friendly materials. It is biocompatible, biodegradable, and possesses good mechanical strength and flexibility [149,150,151]. Additionally, it is eco-friendly, renewable, and low-cost [130,131,149,150,151,152,153,154]. Consisting of a linear and unbranched homopolysaccharide composed of β-D-glucopyranose units linked by β-1,4 glycosidic bonds [17,47], cellulose is readily available, with wood being the primary natural source, but it is also found in plants such as cotton and flax, as well as in vegetables, fruits, and biowaste [47,149,150]. In the majority of biowaste materials, cellulose forms fibril structures enveloped within a matrix composed of lignin and hemicellulose. Cellulose exhibits some limitations, notably low solubility in both water and most organic solvents due to the presence of strong inter- and intramolecular hydrogen bonds and van der Waals forces [149,150]. Moreover, cellulose is challenging to hydrolyze, and both acidic and enzymatic hydrolysis (cellulase) can result in the decomposition of the cellulose molecules [150].

Cellulose can also be produced by seaweed, fungi, and bacteria [36,47]. Bacterial cellulose (BC), as the name suggests, originates from bacteria [16,46].

BC is chemically equivalent to vegetal cellulose. However, it does not contain by-products such as hemicellulose, pectin, and lignin. BC is obtained through fermentation, and any secondary metabolites, nutrients, and other substances it may contain are easily removed [47,150]. Unlike vegetal cellulose, BC does not require purification because it is already obtained in a pure form (with a high degree of purity), allowing for its nearly direct use [46,47].

Other advantages of BC over vegetal cellulose include high porosity, high water absorption capacity (due to a high number of available hydroxyl groups), a higher degree of crystallinity, mechanical robustness, and permeability to gases and liquids. Despite having a molecular formula similar to that of vegetal cellulose, BC exhibits significant differences in its physical and mechanical properties. BC forms cross-linked networks of fibrils that are 100 times finer than those derived from vegetal cellulose, imparting higher elasticity, flexibility, resistance, crystallinity, and surface area to the 3D network [47,150].

Therefore, BC is a natural hydrophilic polysaccharide that is highly biocompatible, biodegradable, permeable, flexible, non-toxic, non-carcinogenic, and hypoallergenic. It possesses a high degree of native purity, high porosity, crystallinity, and the ability to retain large quantities of water. BC also has the potential for chemical modification [8,16,46,47,155]. The two most commonly used techniques for modifying and optimizing the morphological, mechanical, physical, and chemical characteristics of BC and incorporating components like antibiotics are ex situ solution impregnation, which involves the physical absorption or impregnation of molecules into the pure BC chains (without any modification), through the formation of hydrogen bonds due to the presence of hydroxyl groups in BC. Another technique is in situ modification, which involves the incorporation of molecules (included from the beginning in the culture medium) during the synthesis process of the BC fibril network itself [46].

The properties of BC make it suitable for use as a wound dressing. Its dense nanofibrillar network can retain an appropriate amount of moisture in the wound area while absorbing excess exudate [36,46]. The application of BC in hydrogels has gained prominence due to the 3D arrangement of the BC nanofibrillar network. In addition to its similarity to the ECM, its high surface area and porosity provide support for cell proliferation [8,16,46,47]. It is flexible, soft, and easily removable without interfering with wound healing [7,8,13,16,46,53]. Furthermore, it accelerates granulation [16,47], promotes the re-epithelialization process, and serves as a matrix for tissue regeneration, reducing pain and healing time [16,156,157]. Additionally, it exhibits excellent mechanical behavior, similar to synthetic polymers [8,16]. The high mechanical strength, combined with great flexibility, allows cellulose-based dressings to conform to the wound area [47]. Studies have demonstrated that BC stimulates wound re-epithelialization, efficiently improving wound healing. However, it does not possess antibacterial properties [46].

In vitro studies have demonstrated that culturing BC near cells, such as human umbilical vein endothelial cells, adipose stem cells, and fibroblasts, does not alter their morphology or proliferative capacity [36].

Kwak et al. [155] prepared bacterial cellulose membranes (BCM) for the treatment of second-degree burns in Sprague-Dawley (SD) rats. The rats were divided into two groups: one was treated with BCM, and the other with gauze. The results showed that compared to the gauze-treated group, rats treated with BCM exhibited a thicker epidermis and dermis, more blood vessels, reduced mast cell infiltration, decreased expression of VEGF and angiopoietin-1, and increased collagen expression. Additionally, metabolic enzymes indicative of kidney and liver toxicity remained at normal levels. These findings suggest that BCM can enhance the burn healing process by regulating angiogenesis, re-epithelialization, and stimulating connective tissue formation. Furthermore, they did not induce specific kidney or liver toxicity.

#### 4.1.7. Dextran

Dextran is a linear, neutral homopolysaccharide composed of repeated D-glucopyranose units, primarily linked by α-1,6 glycosidic bonds. Additionally, it may include branching α-1,2, α-1,3, and α-1,4 linkages [54,158,159,160,161,162,163,164,165,166,167].

Dextran can be produced by various lactic acid bacteria and results from glucose condensation through the activity of a secreted dextransucrase enzyme, which transfers glucose components from sucrose and synthesizes dextrans with different structures, molecular weights, linkages, and branching patterns, depending on the bacterial genus [54,160]. Dextran produced by *Leuconostoc mesenteroides* contains about 5% of α-1,3-glycopyranosidic linkages, while that extracted from *Weissella* strains has a highly linear backbone with only 3–4% α-1,3 branching [49].

This polymer is hydrophilic, biodegradable, highly biocompatible, non-toxic, and non-immunogenic [158,159,160,161,162,168,169,170]. In addition to being sustainable, it is safe [171] and eco-friendly [133,172,173], and is well known for its low cost and wide natural abundance [130,160,161,162,167,173].

Lately, dextran-based hydrogels have attracted significant attention as wound-healing dressings due to their biocompatibility and flexible and soft characteristics [169]. In addition, this naturally derived material is promising for soft tissue engineering due to its similarity to the native ECM [158]. It can provide nutrients and essential elements for tissue regeneration and support cell proliferation [168].

It possesses excellent water solubility [163,167,170]. However, the high level of branching (α-1,2, α-1,3, and α-1,4 linkages) negatively impacts its solubility. It has been reported that dextrans with more than 43% of α-1,3 linkages cannot be dissolved in water [48].

This glucose-containing polysaccharide possesses three hydroxyl groups on each glucopyranose unit. These not only provide it with high hydrophilicity and good water binding capacity but also make it available for chemical functionalization and cross-linking [158,160,162,164,165]. In addition to effectively absorbing exudate and maintaining the wound bed with appropriate moisture, dextran hydrogels are transparent, facilitating the monitoring of the healing process [174].

Dextran-based hydrogels can be obtained by incorporating polymerizable groups into dextran to facilitate cross-linking [165]. Moreover, the D-glucopyranose residues can be easily chemically oxidized, resulting in oxidized dextran with aldehyde groups, which, in turn, can react with polymers such as gelatin and chitosan or their derivatives with amino side groups, forming in situ hydrogels with novel tissue adhesive characteristics and hemostatic and antibacterial properties [159].

It has been reported that dextran hydrogel scaffolds can enhance angiogenic properties. Additionally, they can serve as platforms to incorporate growth factors/drugs for skin regeneration [169]. Dextran binds to glucan-specific receptors present in human fibroblasts, which, in turn, induce the expression of growth factors that promote cell proliferation, accelerating wound healing [158,170,175].

It possesses immunomodulatory functions, and it can activate neutrophils and macrophages, stimulate cytokine secretion, and strengthen the immune system [165]. Moreover, dextran can function as a mild reactive oxygen radicals (ROSs) scavenger and potentially reduce excessive platelet activation [163].

However, pure dextran hydrogels present some limitations, particularly concerning low mechanical strength, weak tissue adhesion, poor drug loading ability, and undesirable degradation, which chemical and physical modifications help to overcome [167,168].

The biodegradation of dextran in the human body is controlled by dextranases [160,162,163]. The modification of the dextran hydrogel provided gradual material degradation, allowing for proper host tissue integration and ensuring complete skin regeneration [158].

Shen et al. [54] prepared a dextran hydrogel for the treatment of third-degree burn wounds in pigs, and the hydrogel stimulated rapid wound closure, improved re-epithelialization and ECM remodeling, and promoted angiogenesis in a third-degree porcine burn model. In another study on third-degree burns in mice, the dextran-based hydrogel exhibited good bioactivity, particularly in enhancing angiogenic responses and facilitating complete skin regeneration in vivo throughout wound healing. In addition, it recruited endothelial progenitors and cells, promoted epithelial cell migration to the wound area, and supported epithelial differentiation [54,158,166,169,170].

#### 4.1.8. Chitosan

After cellulose, chitin is the second most abundant polysaccharide on the planet, containing amino and hydroxyl groups in its composition [51,52]. Chitosan results from the partial deacetylation of chitin [9,16,50,51,52,53]. This process can occur under harsh alkaline conditions, through treatment with sodium hydroxide (chemical hydrolysis), or in the presence of specific enzymes like chitin deacetylase (enzymatic hydrolysis) [50,52].

There are different degrees of deacetylation, which is an important chemical characteristic of this polymer. During the deacetylation process, the acetyl group (–C_2_H_3_O) is replaced by the amino group (–NH_2_) in the polymer chain. Chitosan is a copolymer composed of units of D-glucosamine ((1,4)-2-amino-2-deoxy-β-D-glucan) and N-acetyl D-glucosamine ((1,4)-2-acetamido-2-deoxy-β-D-glucan). To be called “chitosan,” the degree of deacetylation must be at least 60%, which means it has 60% D-glucosamine units and 40% N-acetyl D-glucosamine [50,51,52].

Its solubility depends on the degree of deacetylation and the pH of the solution. Chitosan is soluble in slightly acidic solutions because the pKa value of its amino group is approximately 6.3. When the pH is lower, protonation of the –NH_2_ groups occurs, converting them into –NH_3_^+^, which increases electrical repulsion, resulting in a soluble cationic polymer [50,51,52].

Chitosan is a linear cationic hydrophilic polysaccharide that is highly biocompatible and biodegradable [11,38,46,137]. It is low-cost [98,130,132,134,176,177] and possesses good swelling properties [78,178], providing a moist environment and promoting effective hydration of the wound area [179,180]. Furthermore, it is renewable [130,131] and eco-friendly [67,131,132,133,134,154,178].

It is an oxygen-permeable biopolymer, non-toxic, with excellent bioadhesive characteristics, tissue adhesiveness, and hemostatic properties [11,16,46,50,53,137,181]. In other words, blood coagulation occurs because chitosan, positively charged, binds to red blood cells due to the negative charge on the cell membrane residues, resulting in hemagglutination [46,50]. The higher the degree of deacetylation, the greater the number of positive charges, and thus, the better the tissue adhesiveness and hemostatic properties [52,182]. Hemostatic activity assists in blood clotting and reduces pain by blocking nerve endings [46]. On the other hand, chitosan adsorbs plasma proteins and fibrinogen, resulting in platelet aggregation [183]. Chitosan has anti-inflammatory activity and wound-healing properties, accelerating wound closure [16,46]. Chitosan speeds up tissue regeneration through wound contraction, stimulating cells such as macrophages and fibroblasts [46]. Furthermore, it exerts an immunomodulatory effect, which also contributes to wound healing [52]. Additionally, its structural similarity to the native ECM promotes cell adhesion and proliferation [137].

Moreover, it exhibits intrinsic antibacterial activity [9,11,16,46,52,53,137], which depends on its molecular weight, degree of deacetylation, concentration, and type of bacteria [184]. Several mechanisms have been proposed to elucidate this activity, with the most widely accepted one being that chitosan chains contain amino groups that can be protonated, giving them a positive charge. Electrostatic interactions occur between the protonated amino groups (–NH_3_^+^) of chitosan and the negative charges of the microbial cell membrane, leading to alteration of cell permeability, resulting in cell membrane lysis and, consequently, cell death [46,50,51,129]. Chitosan demonstrated a bacterial inhibition rate of 95.6% for *Escherichia coli* and 99.2% for *Staphylococcus aureus* [184].

Chitosan degradation produces non-toxic residues, and the degradation rate varies with the degree of deacetylation (lower degrees result in higher biodegradation) and the polymer’s molecular weight (shorter chains degrade more rapidly) [50].

Chitosan-based hydrogels can be formed through physical, chemical, or metal ion coordination cross-linking. Chitosan does not require any toxic additives for gelation. Physical bonds, such as hydrogen bonds, electrostatic interactions, or hydrophobic interactions, are reversible and non-covalent. They depend on factors like temperature, pH, and others, making them relatively unstable. The polycationic nature of chitosan, under acidic conditions, allows for the formation of hydrogels through electrostatic interactions with natural or synthetic polyelectrolytes or polyanions. Among natural polyanions, proteins (such as collagen and gelatin) and polysaccharides (like alginate and HA) have been documented. Examples of synthetic polyanions include polyacrylic acid. Hydrogels formed through physical cross-linking are soft, flexible, and non-toxic. However, their main disadvantages include low mechanical strength, the possibility of uncontrolled dissolution (instability), and the difficulty of controlling pore size. Therefore, chemically cross-linked hydrogels have been investigated. Chemically cross-linked hydrogels are much more stable because covalent bonds are irreversible. In this approach, the primary structure (amino groups) undergoes chemical modifications, which can alter some chitosan properties. There may be contamination with catalysts or residues of toxic reagents. Some commonly used cross-linking agents are glutaraldehyde, glyoxal, ethylene glycol, and diglycidyl ether. However, all of these can confer toxicity to the resulting hydrogel. Genipin is a naturally derived cross-linker that has been used as an alternative, but its toxicity is not well-established. Although chemically cross-linked hydrogels may be toxic, this strategy allows for the production of soft, flexible, and stable hydrogels with better control of pore size. In addition to these two strategies, chitosan hydrogels can be formed through coordination complex cross-linking using metal ions such as Pd (II), Pt (II), and Mo (VI). However, they are less suitable for medical use [50].

Carboxymethyl chitosan is derived from chitosan. It is a biocompatible, water-soluble polymer with wound healing, hemostatic, and antibacterial properties [1,129]. In comparison to chitosan, carboxymethyl chitosan has more positive charges, leading to increased electrostatic interactions and, consequently, higher antimicrobial activity [185]. Previous studies suggest that carboxymethyl chitosan exhibits healing properties in vitro and in vivo by activating macrophages, secreting various cytokines, and stimulating fibroblast proliferation [16].

### 4.2. Synthetic Polymers

Synthetic polymers are also widely used in biomedical applications, particularly as wound dressings in the context of healing. Among the most commonly used synthetic polymers are polyurethane, polyethylene glycol, polyvinyl alcohol, poly-ε-caprolactone, poly-N-vinylpyrrolidone, and poly(lactic-co-glycolic) acid. Figure 2 schematically represents these synthetic polymers. 

#### 4.2.1. Polyurethane

Polyurethane (PU) contains repetitive units of the urethane group [54,55]. It is a polymeric resin [186] and has a microstructure comprising soft and hard segments, which imparts antithrombotic properties and excellent mechanical properties [55,187,188]. The soft segment (polyol part) provides better flexibility, while the hard segment (isocyanate part) imparts strength and hardness [189]. It is possible to adjust the physical properties based on the type and content of the segments used in the PU chain, according to the desired final product [190]. For example, the stiffness of the polymer depends on the extent of crosslinking [56].

This synthetic polymer is highly biocompatible [9,39,54,55,60,187,188,191] and possesses versatile features such as high strength, durability, excellent elasticity, and adjustable degradation rates according to the application [39,54,60,190]. In addition to excellent flexibility [9,55,186], PU-based hydrogels also exhibit great transparency, good adhesion, high mechanical and tensile strength, fatigue resistance, and toughness, similar to biological soft tissues [186,192,193]. PU hydrogels are non-toxic, easily modifiable [193], provide good gaseous permeability, and are more economical compared to natural polymers [9].

PU and its derivatives are generally considered biodegradable, with few exceptions [60]. The selection of synthesis compounds for PUs enables control over their degradation rate [191,194]. It forms highly porous and permeable hydrogels [188], with ease of processing and the ability to modify surface functional characteristics [188,190]. Porous tissue scaffolds possess a structure similar to that of the epidermis and are more suitable for skin regeneration [194]. The ability to control pore size and polymer chemical properties allows for the regulation of drug release rates. Therefore, PU-based dressings can be utilized as drug delivery systems [195]. 

It is a widely used polymer in the biomedical field, especially in wound dressings [187,188,190]. It promotes cell proliferation, and its matrix allows for the regeneration of deeper skin layers such as the dermis [11]. However, it has some limitations, such as the absence of antibacterial properties [188,194,195], and the potential for side effects from its degradation products [39].

The primary application of hydrophilic PUs is in wound dressings. The majority of PUs are hydrophobic. However, it is possible to obtain hydrophilic PUs by introducing hydrophilic groups into the soft-segment PU structure, with polyethylene glycol being the most common hydrophilic functional group [196]. It has been reported that PU cannot completely prevent water permeation due to the presence of various polar functional urethane groups in its chain, which could be critical when incorporating batteries from biological moisture or electronic circuits. One solution involves using a highly hydrophobic PU to prevent water permeability and moisture diffusion [190]. The introduction of polyethylene glycol gradients into the PU backbone enables it to absorb water effectively [193].

It is described in the literature that hydrophilic PU foams are highly absorbent and excellent candidates for use as dressings, as they provide a consistently hydrated environment in the wound area [195]. PU dressings are suitable for the management of highly exudative wounds, such as burns, because PUs effectively absorb exudate and maintain wound moisture due to their small and uniform pores. There is a balance between water absorption, moisture retention, and water loss through evaporation that makes PU effective as a wound dressing [195]. Moreover, they are cost-effective and have a simple manufacturing process [197]. In vitro studies conducted on keratinocyte HaCaT cells indicate that PU foams are non-cytotoxic [195]. Similarly, hydrophilic porous PU sponges exhibit high water absorption and moisture retention capacity, as well as good gas permeability. They are non-cytotoxic and cost-effective [197,198].

Little is known about the tissue adhesion properties of PU-based hydrogels. Wen et al. addressed this by adding tannic acid to the PU-polyethylene glycol hydrogel. Tannic acid, with its chemical structure containing multiple catechol groups that bind to urethane groups and form hydrogen bonds, imparts good adhesion to the PU-polyethylene glycol hydrogel [199].

Although PU is not considered eco-friendly, research has been conducted to develop more environmentally friendly alternatives [200,201]. The isocyanates commonly used in the production of most PUs typically derive from the reaction of amines with highly toxic phosgene compounds [55]. Moreover, the isocyanates themselves are toxic and have been associated with occupational asthma [202] due to their high volatility and the tendency to convert in vitro into aromatic diamines, compounds that are toxic, carcinogenic, and/or mutagenic to humans. Even in minimal amounts, there is a possibility of these toxic residual compounds leaching from PU-based products [55]. The trend is to replace isocyanates with alternatives that are less toxic and greener [202].

Efforts have been made to minimize toxicity, notably through the synthesis of PU using polyisocyanates derived from amino acids that undergo controlled biodegradation, resulting in non-cytotoxic byproducts. However, there is still the possibility of unreacted polyisocyanates remaining in the final product [55]. In an attempt to develop alternatives that do not involve the toxic chemistry of isocyanates, cationic, anionic, and neutral PU hydrogels were prepared through a non-isocyanate route under mild conditions, without the need for catalysts. Hydroxyurethane functions were obtained through the aminolysis of five-membered cyclic carbonates. This approach is safer and more environmentally friendly [202]. Chen et al. [203] prepared a water-borne PU nanofibrous membrane using water in its preparation instead of organic solvents, eliminating environmental and toxicity concerns. The membrane was biocompatible, non-toxic, and biodegraded within 4 to 6 weeks. Waterborne PU is easily functionalized due to its numerous functional groups (–COONH^−^ and –COO^−^), allowing it to interact with other chemicals effectively [204]. It has been widely used as an adhesive. It offers several advantages, including good toughness, strong mechanical properties, wear resistance, and excellent adhesion [205]. Additionally, waterborne PU is eco-friendly [189,205,206,207,208].

#### 4.2.2. Polyethylene Glycol

Polyethylene glycol (PEG) is a synthetic polyether [54,209] that can have linear or branched structures, including multi-arm or star configurations. The basic structure of PEG comprises a PEG diol with two terminal hydroxyl groups. These groups can be converted into various other functional groups, such as amine, carboxyl, vinyl sulfone, thiol, acetylene, acrylate, and aldehyde groups, enabling their conjugation with other materials [209,210].

PEG is considered harmless to the human body [58], and is a recognized eco-friendly polymer [211]. It is highly biocompatible, non-toxic, non-immunogenic [17,41,54,60,209,212,213,214], a low-cost polymer [215,216], with good water solubility [17,41,60,212,213], flexibility [60], great permeability [217], and adjustable mechanical properties [209,218]. In addition to being soluble in water, it is also soluble in various organic solvents, such as ethanol and acetone [214]. Moreover, PEG demonstrates good large-scale production capabilities, and hemocompatibility [219].

PEG is often referred to as a “stealth material” [220], due to its resistance to protein adsorption [17,39,40], and it generally resists cell attachment because of its higher affinity for water binding [221]. Regarding its tissue adhesion capability, little is known about PEG on its own. However, Krishnadoss et al. [222] incorporated choline molecules into synthetic polymers such as PEG to enhance their adhesive strength. Another group of researchers reported that PEG alone cannot adhere to tissue wounds; however, PEG derivatives, specifically polyethylene glycol diacrylate, have been utilized as tissue glue, owing to their capacity to adhere to both skin and injured tissues [223].

Studies have shown that PEG can improve the mechanical strength, stability, and degradation rate of hydrogels [54,57,209,210]. However, it has also been reported that PEG hydrogels are brittle and weak [223]. Furthermore, the ease of controlling its chemical composition and scaffold architecture makes this polymer attractive for tissue engineering applications [40]. PEG-based hydrogels exhibit very small variations between batches, providing robust and reproducible substrates [218]. Some authors consider PEG as biodegradable [54,58,214,223], while others assert that it is non-biodegradable [39,40].

This polymer has been studied in the context of wound healing due to its high biocompatibility, transparency, and ability to provide support for healing [41,54,224]. It is a hydrophilic material capable of maintaining a moist environment while absorbing wound exudate [19,30,212]. PEG hydrogels exhibit a high swelling rate [223], allow for gaseous permeation [30], eliminate bacteria and prevent their proliferation [2]. Furthermore, PEG hydrogels can also be utilized as drug delivery systems and for cell encapsulation [218,225].

PEG has some limitations, notably its tendency to swell and undergo oxidative degradation in aqueous environments, compromising its mechanical durability and limiting its long-term effectiveness. Additionally, PEG lacks antibacterial properties [226]. Furthermore, due to their bioinert nature, PEG hydrogels alone are unable to provide an optimal environment to support cell adhesion and tissue formation [98].

#### 4.2.3. Polyvinyl Alcohol

Polyvinyl alcohol (PVA), an eco-friendly polymer [86,227,228,229], is commonly used as a wound dressing in the treatment of cutaneous wounds [11,40,54]. Structurally, it is a vinyl polymer interconnected through carbon–carbon bonds. The presence of hydroxyl groups in the side chains allows PVA to self-crosslink [40,230].

PVA is a synthetic polymer that is highly biocompatible, biodegradable, and hydrophilic, with high water affinity [17,39,60,119,230,231,232,233,234,235]. It is easily processed [231], possesses good water solubility [17,40,60,230,233,234,236], high crystallinity, and pH sensitivity [39,60]. Additionally, it has a low tendency for protein adhesion [59,119], good chemical stability, and is toxicologically harmless [40,236], non-carcinogenic, non-toxic, and cost-effective [17,59,215,233,237]. PVA possesses desirable physical and mechanical properties, including good gas permeability [17], and other favorable physical properties such as a rubbery nature, high expansion index, high water swelling degree, fluid absorption ability, and good transparency [59,233].

PVA is a promising candidate for the preparation of biomedical hydrogels, typically prepared by physical crosslinking [236]. PVA-based hydrogels can be prepared without the addition of a chemical crosslinking agent, through freezing–thawing methods, and are simple, safe, and non-toxic [238]. However, it was experimentally observed that pure PVA hydrogels prepared through the cyclic freezing–thawing process exhibited relatively weak mechanical performance, attributed to the limited number of crosslinking agents in the formed polymeric network. Consequently, the combination of PVA with another polymer is necessary to enhance its mechanical performance [214].

PVA hydrogels can also be formed using chemical methods and irradiation, among others [234]. For instance, Oliveira et al. [3,239,240,241] prepared a PVA hydrogel through gamma irradiation for burn healing and loaded it with silver nanoparticles, resulting in antimicrobial hydrogels effective against *S. aureus*, *E. coli*, and *Candida albicans*. PVA hydrogels are permeable to small molecules [242]. Moreover, characteristics such as transparency, mechanical resistance, biocompatibility, biodegradability, and the ability to maintain a hydrated environment and ensure structural stability when hydrated make PVA-based hydrogels favorable for treating wounds such as burns [54].

There is some controversy regarding certain properties of PVA, particularly its mechanical properties and tissue adhesion. Some authors argue that the polymer possesses good mechanical strength, considering it to be similar to cutaneous tissue [232,236], and it has even been described in the literature that PVA is often used to enhance the mechanical performance of other polymers in wound dressings [59], while others claim that its mechanical properties are weak [234,243,244]. Regarding adhesive properties, some authors state that PVA has good adhesive properties [235], while others assert that it exhibits weak tissue adhesion [234].

PVA has some limitations that restrict its use as a wound dressing, such as a lack of antibacterial properties [234], low elasticity, and poor adhesion to cells and tissues [11,231,234]. Some authors not only argue that PVA’s elasticity is inadequate but also consider its hydrophilic characteristics to be incomplete, and its membrane to be rigid. Therefore, it needs to be combined with other polymers to overcome these limitations [11,59,234].

There are studies in which PVA was combined with chitosan [245], chitosan and honey [230], chitosan and gelatin [232,233], or BC [234] that have shown promise. Shamloo et al. [232] prepared a PVA-based hydrogel with chitosan and gelatin and found that increasing the PVA concentration improved the encapsulation efficiency, slowed the degradation rate, and reduced the drug release rate. Chopra et al. [230] developed a hydrogel based on PVA and chitosan that was physically cross-linked without the use of organic solvents or harmful chemicals.

#### 4.2.4. Poly-ε-Caprolactone

Poly-ε-caprolactone (PCL) is a semi-crystalline linear petroleum-based polyester [39,54,60,237,246]. This aliphatic polymer [40,56,246] is eco-friendly [247,248], non-toxic, non-immunogenic, and has good biocompatibility and biodegradability. However, its degradation rate is slower than other polyesters [39,237,246,249], making it less appealing in biomedical applications. However, it is more attractive for long-term implant applications or controlled release systems [59,237]. Currently, there are strategies to accelerate the biodegradability of this polyester. This includes incorporating a more reactive hydrolytic group into the structure, using more hydrophilic and acidic end groups, or employing PCL with a lower molecular weight [250].

Additionally, it has advantageous properties such as excellent mechanical properties, high toughness, elongation, good elasticity, and flexibility [40,54,60,237,251]. Its hydrophobic nature makes it water-resistant and less prone to swelling, and it is also minimally permeable to water vapor [252]. It is cost-effective [40,253,254]. It possesses a low melting point (55–65 °C) [40,54,246,249,254], enabling the easy processing of PCL through a variety of techniques [254], and a glass transition temperature at −60 °C [40,54,246]. Moreover, PCL exhibits high physical stability, simple preparation methods [54,253], remarkable blend compatibility, good solubility in many solvents, and high ductility and plasticity, which facilitate its handling and conformation. Furthermore, PCL dressings are semi-permeable and occlusive, and are easy to remove, because they are non-adherent [246]. Moreover, it is possible to control the mechanical properties, geometry, fiber, and pore size, among other properties, of PCL scaffolds [250]. PCL is suitable for low-temperature 3D printing [255].

It is widely employed in biomedical applications [249], especially in the context of wound healing and tissue regeneration, owing to its properties of reducing inflammatory infiltration and promoting rapid wound healing [59], and as a drug delivery system for controlled release [54]. Features such as low toxicity, slow biodegradation [256,257,258], and great permeability to many drugs allow PCL to be used as microcarriers for the administration of active compounds over extended periods [217]. Studies indicate that PCL can remain intact in the body for long periods before being fully metabolized and excreted. The low degradation rate is attributed to the presence of five CH_2_ groups in the polymer’s composition, making it hydrophobic and less susceptible to hydrolysis [258]. Its degradation by-products are non-toxic [255]. Moreover, short- and long-term biocompatibility studies were conducted in animal models, and no adverse effects were observed with PCL scaffolds over 2 years [250].

However, its application in tissue engineering is primarily limited by its high hydrophobicity [39,40,60,249], as well as low bioactivity. This limitation arises from the absence of functional groups and proper cell recognition sites on the scaffold’s surface [40,54,249,255]. One possible solution to address these issues is to combine PCL with other polymers [40,54,249,253]. On the one hand, the high hydrophobicity significantly limits PCL’s ability to absorb wound exudates [253]. Partial hydrolysis of ester linkages does not compromise the mechanical properties and increases the concentration of free hydroxyl and carboxyl groups, enhancing the hydrophilicity and water binding capacity of PCL [253]. Polymers such as PEG create more hydrophilic structures, exhibiting improved mechanical properties and degradability [40,54,259]. Therefore, by moderately increasing hydrophilicity, the exudate absorption capacity of PCL-based dressings would be enhanced, which is highly desirable in wound healing processes [253]. On the other hand, the lack of functional groups that facilitate cellular adhesion reduces its cellular affinity, compromising tissue regeneration [249,253]. Gelatin/PCL scaffold allows for leveraging the suitable mechanical strength of PCL and capitalizing on the positive aspects of gelatin, including good biodegradation, cell adhesion, and proliferation [249]. Another drawback of PCL is the lack of antibacterial activity, which can be addressed by loading the PCL dressing with antibacterial drugs [59].

The ester linkage of PCL is prone to hydrolytic degradation by lipase enzyme, an ester-like enzyme. Bacteria-producing lipase could be employed for specific and localized delivery of antibacterial drugs at the infection site [260].

Gupta et al. [261] prepared a PEG-PVA-based hydrogel for burn treatment. The hydrogel exhibited high elasticity, good long-term stability, robust mechanical strength, and a favorable water vapor transmission rate. Additionally, it demonstrated a significant capacity for absorbing bodily fluids (in vivo), resulting in enhanced healing efficiency. 

#### 4.2.5. Poly-N-Vinylpyrrolidone

Poly-N-vinylpyrrolidone (PVP) contains a hydrophobic polymeric chain and a hydrophilic amide carbonyl group in its structure [262].

This synthetic polymer is widely used as a skin alternative product since it does not cause skin irritation [40]. It offers several beneficial features, including good biocompatibility, biodegradability, low cytotoxicity, excellent water vapor transmission, environmental stability, and high thermal and chemical resistance [17,39,40]. Furthermore, PVP is eco-friendly [263] and demonstrates good molecular control, large-scale production capabilities, and hemocompatibility [219].

Due to its molecular structure, PVP is water-soluble and exhibits very good solubility in most organic solvents, as well as good affinity to complex hydrophobic and hydrophilic substances [17,39,40,262].

The adhesive properties of PVP surpass those of PEG. While PEG relies on hydrogen bonds provided by repeated ether groups for adhesion, PVP offers tertiary amides that provide multiple sites for hydrogen bond formation, significantly enhancing adhesive properties [264]. Moreover, it is low-cost [263] and possesses hemocompatibility, temperature resistance, and pH stability [17].

This hydrophilic polymer has been employed in drug delivery systems and tissue engineering [262], and in the biomedical field for wound treatment, due to its biocompatibility, biodegradability, and low toxicity [265,266]. Moreover, it has been reported that PVP can be used in the treatment of first- and second-degree burns, as well as severe sunburns, in the form of PVP-based hydrogels [54,262].

PVP hydrogels, formed through the crosslinking of PVP under ionizing radiation, resulting in crystal-clear hydrogels with outstanding transparency [54,262]. However, there is no consensus regarding their swelling capacity. Some authors claim that the properties are good [262], while others assert that the swelling capacity of these hydrogels is limited [54]. In vitro and in vivo studies suggest that PVP biomaterials obtained through ionizing radiation did not exhibit toxic effects and are considered safe. Therefore, they can be used in contact with the skin, such as wound dressings [262].

The mechanical properties also generate some controversy. On the one hand, some authors argue that PVP has good mechanical properties [266,267]. On the other hand, some authors contend that its mechanical properties are weak [54], and PVP needs to be mixed with polysaccharides [54] or hydrophilic plasticizers such as PEG to overcome these limitations [268]. Additionally, the combination of PVP with other polymers allows it to acquire useful characteristics for wound healing, such as the ability to prevent microbial penetration [40,54].

#### 4.2.6. Poly(lactic-co-glycolic) Acid

Polylactic acid (PLA) is an aliphatic polyester that exhibits excellent biocompatibility, biodegradability, and good integrity, along with tunable mechanical properties, making it suitable for various biomedical applications [9,39,40,60,237]. Moreover, PLA is hydrophobic, non-toxic, chemically inert, structurally highly stable, and inexpensive [9,39,40,60]. Unlike some natural polymers, PLA can be used on contaminated wounds as it effectively inhibits the propagation of bacteria [237]. PLA and its copolymers are considered eco-friendly [237,269]. The ester backbone is naturally biodegraded through enzymatic action or hydrolysis under physiological conditions. This process leads to non-harmful and non-toxic products that are easily absorbed by the body through natural metabolic pathways [39,40,56]. Its degradation can be properly controlled, and it is relatively moderate [237]. PLA can be employed as drug delivery systems, with low-molecular-weight PLAs degrading more slowly and proving more efficient in drug release [237].

Polyglycolic acid (PGA) is harmless, non-toxic [270], and eco-friendly [270,271]. PGA is another linear aliphatic polyester. This polymer is biodegradable and biocompatible, with high tensile strength and excellent mechanical properties. While more hydrophilic than PLA, PGA is rapidly biodegraded through hydrolysis, yielding glycolic acid. Excessive production of glycolic acid leads to a decrease in pH in its microenvironment due to carbon dioxide production, resulting in local necrosis of cells and tissues. Additionally, it can trigger an inflammatory response [39,40,251]. In addition to tissue inflammation, PGA can induce a severe foreign body reaction [237]. Moreover, the glycolic acid released from PGA may exhibit bacteriostatic properties [237].

Poly(lactic-co-glycolic) acid (PLGA) is a linear aliphatic polyester copolymer that possesses good biocompatibility, biodegradability, easy handling, and mechanical strength [40,54,57,60]. It is also eco-friendly [272] and exhibits weak tissue adhesion [237]. PLGA is relatively hydrophobic [56,237], making it soluble in organic solvents such as acetone, ethyl acetate, tetrahydrofuran, and chlorinated solvents [56]. If the polymer contains less than 70% PLA, it is considered unstructured [56]. Its glass transition temperature is between 40 and 60 °C, and it undergoes hydrolysis in the body [60], and its hydrolysis products can be uptaken in the cellular metabolic pathway [57]. The hydrolysis of PLGA results in the formation of natural metabolic monomers (salt form of lactic acid and glycolic acid), which are relatively harmless, minimizing the risk of systemic toxicity [273]. The degradation rate can be adjusted by varying monomer ratios [9,39]. The healing process occurs within a specific time frame and depends on the synchronization of the epithelialization rate with the degradation rate of PLGA [54]. In vitro studies have shown that PLGA exhibits excellent cytocompatibility in fibroblasts, with minimal toxicity [237]. It is widely used in tissue engineering and as a drug delivery system, primarily due to its beneficial characteristics such as adjustable and controllable mechanical properties [40,54,237,273,274]. It can transport both hydrophobic or hydrophilic drugs, small or macromolecules [56,273], and protect them from degradation and control their release [273]. PLGA films are hydrophobic, stiff, and semi-permeable. They lack the ability to absorb exudates or provide a humid microenvironment. On the other hand, PLGA nanofibers are highly dense, allowing them to prevent bacterial invasion. PLGA possesses characteristics that make it suitable for use as an outer layer, capable of preserving moisture content and isolating a hydrogel matrix from the external environment [274]. Wang et al. [274] prepared a bilayer membrane scaffold using 3D printing, consisting of an outer layer made of PLGA and a lower layer of alginate hydrogel, mimicking the epidermis and dermis, respectively. The multiporous alginate hydrogel was employed to enhance in vitro cell adhesion and proliferation, while the PLGA membrane served to prevent bacterial invasion, reduce evaporation, and maintain the moisture content of the hydrogel.

## 5. Overview of Critical Attributes of Polymers

Table 3 compiles key information on critical attributes of polymers as candidates for the ideal wound dressing. The terms used in the table, though relative, aim to facilitate the interpretation of individual properties of each polymer and enable comparison. To the best of our knowledge, the literature lacks comprehensive information on all the features we consider critical for all polymers, both individually and in the form of hydrogels. The table presents a relative critical analysis on a scale from “-” to “+++” where “-” indicates the polymer lacks a specific property, and “+++” signifies excellent performance.

It should be noted that regarding moisture, the literature does not clearly distinguish between the different abilities of hydrogels to provide a hydrated environment in the wound bed. Similarly, concerning transparency and cost-effectiveness, there is also no clear distinction among polymers in the literature. Regarding protection, although it is generally known that hydrogels, to some extent, prevent the entry of microorganisms by forming a physical barrier between the wound and the external environment, here, we aim to convey the capacity for protection in terms of having intrinsic antibacterial properties. Regarding safety, we considered the concept of “non-toxic”. A more in-depth discussion of this issue may be necessary. Finally, concerning the availability of synthetic polymers, since they are chemically synthesized, it was not considered. In addition to the commonly considered critical quality attributes for an ideal wound dressing, we also considered the environmental aspect and added a column on the environmental sustainability of the polymers.

## 6. Applications of Hydrogels as Wound Dressings

**Figure 3:**
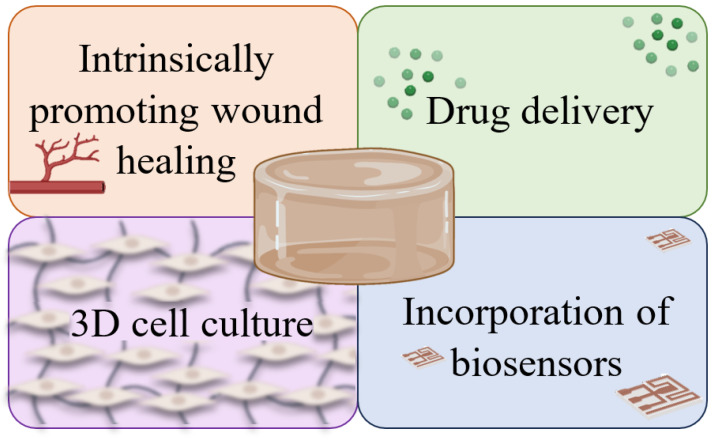
Representation of some of the applications of hydrogels as wound dressings: intrinsic capacity to stimulate healing; drug delivery systems or transporters of other substances; support for cell growth; real-time monitoring of the state of wounds through the incorporation of biosensors.

Hydrogels are promising candidates for the treatment of cutaneous wounds and can be approached in different ways given the potential of their characteristics. Some of the applications of hydrogels as dressings include promoting wound healing through their inherent properties, delivering substances such as drugs and growth factors, enabling cell growth within their 3D structure, which mimics the native structures of the skin, or, more recently, incorporating biosensors for real-time monitoring of wound characteristics. Figure 3 provides a schematic representation of these applications.

### 6.1. Hydrogels with the Intrinsic Ability to Promote Wound Healing

**Figure 4:**
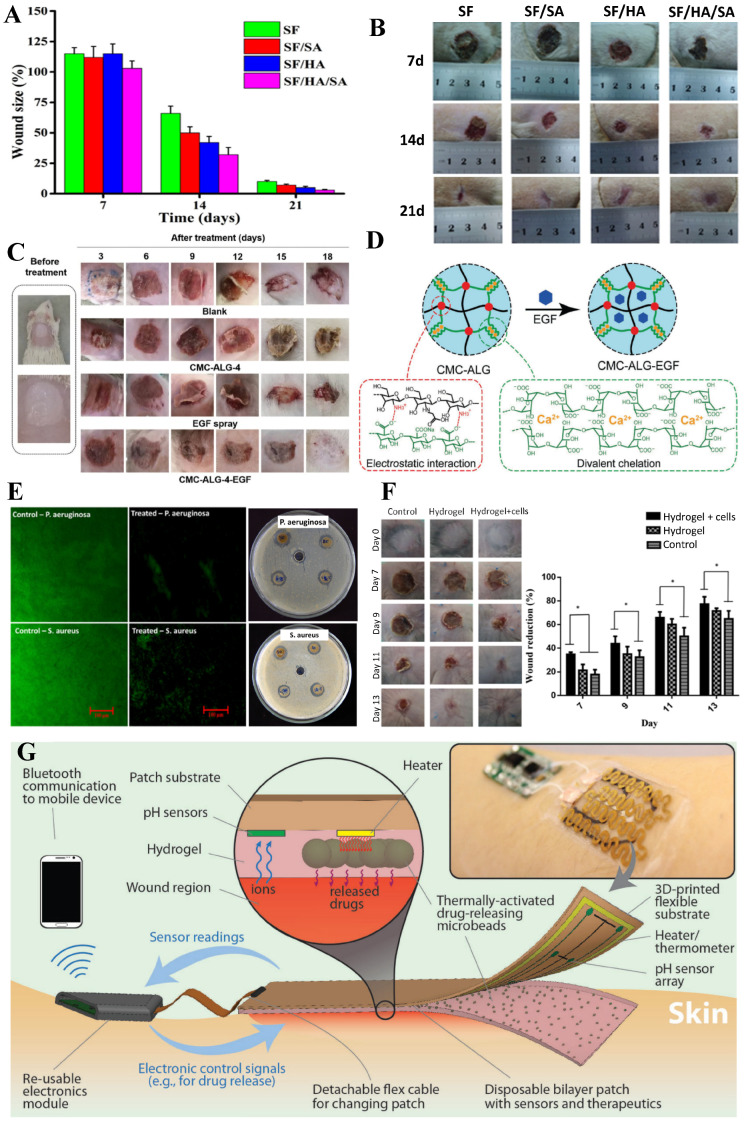
(**A**) Comparison of wound size over 21 days after treatment with hydrogels. (**B**) In vivo observation of burn wound healing over 21 days. (**C**) In vivo study on EGF delivery and wound healing promotion. (**D**) Schematic representation of EGF loaded in carboxymethyl chitosan and alginate hydrogel. (**E**) In vitro inhibition of *S. aureus* and *P. aeruginosa* growth after loading ciprofloxacin in SF hydrogel. (**F**) In vivo study on partial-thickness burns treatment with hydrogels loaded with keratinocytes and fibroblasts (* means significant difference (*p* < 0.05) between the groups) (**G**) Schematic representation of the operation of a hydrogel incorporated with poly (N-isopropyl acrylamide) stimuli-responsive particles. Adapted from: [8,109,276,277,278].

Hydrogels can promote wound healing through their intrinsic characteristics, notably by providing an appropriate cell-friendly microenvironment, stimulating angiogenesis, a key factor for tissue regeneration, or, for example, recruiting cells involved in the healing process [2,3,11]. For instance, Sun et al. [275] prepared a hydrogel based on dextran and PEG for the treatment of third-degree burns in mice. The results show that the hydrogel’s structure allows for the infiltration of neutrophils, which, in turn, facilitates the degradation of the hydrogel during the repair phase. This results in the recruitment of endothelial progenitors and angiogenic cells, stimulating rapid in vivo neovascularization after one week of treatment. Epithelial repair was observed within 14 days, with complete skin and appendage regeneration (sebaceous glands and hair follicles) noted by the end of 21 days. Another example is the hydrogel prepared by Yang et al. [109], composed of SF, HA, and alginate. SF serves as the primary matrix for tissue formation, providing mechanical support and promoting cell adhesion and proliferation. HA enhances biocompatibility, angiogenesis, and tissue regeneration, while alginate improves biocompatibility and hydrophilic performance (Figure 4A). The hydrogel mimics the structure of the ECM in native tissue. This 3D porous microstructure possesses soft and elastic characteristics, along with good physical stability in environments simulating bodily fluids, ensuring adequate mechanical performance. These features enable the adhesion, growth, and proliferation of NIH-3T3 fibroblasts in vitro. In vivo (Figure 4B), the hydrogel creates a favorable environment for healing, facilitating enhanced re-epithelialization, increased collagen deposition, ECM remodeling, and improved angiogenesis, thereby accelerating the burn wound healing process. 

### 6.2. Hydrogels as Drug Delivery Systems and Other Substance Carriers

Furthermore, the matrix of hydrogels allows for the incorporation of substances, such as growth factors, cytokines, or drugs, namely, antibacterials and anti-inflammatories, that assist in the healing process [3,11]. Numerous studies focus on incorporating growth factors into hydrogels to expedite skin healing, such as β-FGF, primary promoters of cell proliferation with chemotactic activity, and VEGF, which enhances cell proliferation and tissue remodeling in wounds [4]. Similarly, in the study conducted by Hu et al. [177] (Figure 4C), they loaded EGF into a carboxymethyl chitosan and alginate hydrogel to protect the EGF from proteolytic degradation (ensuring its bioactivity) (Figure 4D) and to allow for its gradual release, improving cell proliferation. Dong et al. [278] prepared an SF hydrogel loaded with ciprofloxacin for the healing of deep partial-thickness burns in rats. The results showed that the hydrogel effectively delivered the antibiotic, inhibiting in vitro bacterial growth and biofilm formation of *S. aureus* and *P. aeruginosa* (Figure 4E). In addition, the hydrogel promoted autolysis, a reduction in inflammation, fibroblast proliferation (attributed to the silk protein environment), increased collagen deposition, accelerated re-epithelialization, formation of granulation tissue, stimulation of angiogenesis, and showed complete reconstitution of the epidermal layer in rats after 18 days. In the study conducted by Yin et al. [114], they also prepared an SF-based hydrogel, but this time loaded it with rhein to simultaneously prevent bacterial colonization/infection and reduce inflammation. Rhein is a bioactive anthraquinone isolated from the traditional Chinese medicine rhubarb that possesses good anti-inflammatory and antibacterial properties. By incorporating it into SF hydrogels, the stability and structural integrity of rhein might be improved, thus enhancing therapeutic efficacy and minimizing negative effects. The SF/Rhein composite hydrogels combined the excellent biocompatibility and physicochemical properties of SF, along with the antibacterial and anti-inflammatory efficiency of rhein, accelerating the bacterially infected burn wound healing rate by reducing inflammation, expediting angiogenesis, and promoting skin appendages formation. 

### 6.3. Hydrogels as 3D Scaffolds for Cell Adhesion and Proliferation

Hydrogels can serve as a platform for loading cells [3,11]. The 3D matrix of hydrogels allows for the deposition and organization of cells [8], mimicking the environment of the natural biological ECM better than two-dimensional substrates [279,280,281]. The architecture of the hydrogel, characterized by suitable biocompatibility, morphology, and mechanical behavior, enables it to function as a temporary support, facilitating cellular processes such as adhesion, proliferation, and differentiation for the formation of new tissue [282]. The hydrogel can be loaded with cells such as keratinocytes and fibroblasts, as demonstrated in the study by Mohamad et al. [8], where they loaded keratinocytes and fibroblasts into a hydrogel based on BC and acrylic acid. The results were very promising, achieving complete re-epithelialization of a partial-thickness burn within 13 days (Figure 4F). Furthermore, there was a more organized deposition of type I collagen fibers, attributed to the synergistic effect of the hydrogel with the incorporated cells in accelerating skin regeneration and strengthening the dermis. Additionally, the incorporated cells can be stem cells. It is considered that stem cells promote healing through differentiation into specific cell types or through paracrine effects to stimulate the host tissue regeneration [19]. Stem cell therapies have shown promising outcomes in the context of wound healing [283,284]. Dong et al. [19] demonstrated that the HA and poly (ethylene glycol) hydrogel loaded with adipose tissue-derived stem cells provided an optimized 3D microenvironment, enhancing the therapeutic efficiency of stem cell-based therapies. The hydrogel promoted cell paracrine secretion and increased the expression of pro-angiogenic growth factors and cytokines, such as angiopoietin, VEGF, platelet-derived growth factor, stromal cell-derived factor, contributing to the wound healing treatment in a burn animal model.

### 6.4. Hydrogels with Integrated Biosensors

Moreover, the incorporation of biosensors into hydrogels can solve various challenges associated with wounds, such as the early detection of infections and the acquisition of relevant information about the wound microenvironment in real-time. This information can be used to provide timely and accurate reports on the evolution of the healing process [9,11]. Villanueva et al. [285] developed a smart antimicrobial wound dressing based on keratin hydrogels with zinc oxide nanoparticles (nZnO), taking advantage of the pH-responsive behavior of keratin and the antimicrobial activity of nZnO. Infected wounds exhibit alkaline pH due to the by-products of bacterial metabolism. As the wound undergoes healing, the pH becomes acidic. In a clean wound, the dressing acts as a barrier, isolating the injury from the external environment and protecting it from microbial contamination. In a bacterially contaminated wound, the increased pH leads to hydrogel swelling, increasing its pore size, and facilitating the release of the antimicrobial agent into the medium, thereby controlling the infection. Mostafalu et al. [276] developed an alginate hydrogel sheet incorporated with poly (N-isopropyl acrylamide) stimuli-responsive particles (a drug-releasing system), loaded with cefazolin for real-time monitoring of the wound environment for individualized treatment of chronic wounds (Figure 4G). This automated, smart, flexible wound dressing comprises pH sensors and a microheater to trigger thermo-responsive drug carriers containing antibiotics. The pH sensors are connected to a microcontroller through an electronic module that processes the data measured by the sensors. Once the pH exceeds an acceptable range, it communicates wirelessly in a closed-loop manner to smartphones or computers to remotely activate the heater and program the on-demand release of the antibacterial drug.

## 7. Future Prospects

As we envision the future of biomedical research, particularly in the field of hydrogel polymers, numerous promising opportunities emerge, prepared to revolutionize wound healing and tissue engineering. We will witness an intensified focus on the research and development of innovative biomaterials, with an emphasis on sustainability and environmental friendliness. Currently, the concept of sustainability is a foundational pillar across all scientific domains. The increasing demand for environmentally friendly materials has driven research towards the exploration and development of biopolymers derived from natural raw materials that are eco-friendly and sustainable. Thus, biomass-based materials have gained prominence as candidates for applications that leverage renewable resources and address environmental concerns [149,150]. Cellulose, in particular, is a biopolymer that is not only biocompatible, biodegradable, and non-toxic but also renewable, as it is present in the cell walls of a large number of plants [150]. However, further studies should be carried out to explore more alternatives to polymers. Additionally, more research is needed for the development of eco-friendly polymer synthesis and acquisition techniques. It is crucial to clarify the environmental impacts resulting from the methods used to obtain both natural and synthetic polymers. Moreover, the possibility of recycling industrial waste should be considered. As a purely illustrative example, collagen can be extracted from fish scales. With hundreds of millions of tons of fish annually, a significant amount of waste is generated in fish shops and processing factories, where scales constitute a major solid waste. Improper disposal techniques lead to unpleasant odors and environmental pollution. Therefore, it would be worthwhile to explore solutions to optimize the potential of such waste for the production of valuable end products. On the other hand, further in-depth studies on the degradation pathways of polymers, both natural and synthetic, are necessary, as well as the assessment of the potential formation of toxic compounds and the possible risks of degradation products. Due to certain limitations of polymers, most studies involve polymer blends, and the individual potentials and risks of each polymer are not always clear. Therefore, additional research is required to elucidate the effective contribution of polymers to skin wound healing. However, the current well-established knowledge about the effectiveness of hydrogels and their properties implies new challenges. Future research should focus on evaluating their biocompatibility and biodegradability to ensure they are safe for use in patients and degrade naturally without causing environmental harm. The adjustment of the physicochemical properties of hydrogels to achieve the desired outcomes, specific needs, and the characteristics of the patient remains a challenge. In the future, this may be controlled through modifications in the chemical structure of molecules or by selecting molecular weights and types of cross-linking, thus expanding the currently available repertoire of natural sources [34]. The incorporation of bioactive agents promoting wound healing will enable dressings to assume a more active role. Hydrogels capable of releasing drugs in response to specific stimuli could be employed to design active reservoirs for therapeutic cells. The dynamic control of drug release using sensors to determine specific parameters and trigger the release of cells stored in the hydrogel has the potential to enhance both effectiveness and safety, providing a platform for innovative therapeutics [34]. In light of the current understanding of wound dressings, it is anticipated that the future diagnosis and treatment of wounds will be based on a multifunctional and systematic approach, considering the stage and characteristics of the wound [5]. The incorporation of sensors into hydrogels may open doors to real-time wound monitoring without the need for expensive equipment [21]. It is important to highlight a key and pressing aspect for the advancement of the real-world application of these hydrogels: the conduct of robust clinical studies to assess the effectiveness of the hydrogels in different types of wounds, comparing them with conventional treatments. Only this approach will allow for validation of the efficacy of hydrogels and expand their clinical application. 

So, the aspects that will guide the next steps in advancing the development of hydrogels for wound healing will necessarily involve the following approaches: exploration of sustainable biopolymers, recycling of waste, development of multifunctional dressings, controlled drug release, modifications of hydrogel properties, incorporations of therapeutic agents, evaluation of biocompatibility and biodegradability, and performance of robust clinical studies. The focus will be on improving their effectiveness, safety, and environmental impact.

The future of polymers is brimming with possibilities, driven by innovation, sustainability, and improved outcomes for patients. By embracing interdisciplinary collaboration and harnessing cutting-edge technologies, researchers are poised to uncover new frontiers in wound healing and tissue engineering, ushering in an era of unprecedented therapeutic efficacy and environmental responsibility.

## 8. Conclusions

Hydrogels are commonly regarded as one of the most promising wound dressings due to their inherent properties. The literature extensively describes features such as water absorption capacity and wound exudate retention, as well as the mechanical characteristics of each polymer. However, despite the understanding that intrinsic limitations hinder the characterization of polymers in isolation, notably the lack of appropriate mechanical properties, it is paramount to clarify, for each polymer (individually and in hydrogel form), the specific attributes corresponding to the requirements of an ideal dressing. Further studies addressing these specific topics are necessary to elucidate controversies surrounding certain properties and facilitate a more accurate comparison and approach. Despite the existing gaps and the need for additional research, this manuscript can contribute to this process and serve as a foundation for future investigations. These investigations aim to expand the potential of hydrogels as promising wound dressings in biomedical applications, while also considering environmental concerns.

## Figures and Tables

**Table 1:** Critical attributes of an ideal wound dressing.

Critical Attributes	Justification
Moisture	Ensuring a balanced and moist environment to promote cell migration and proliferation. It is considered as key factor for wound healing. (supplying moisture to the dry wounds and absorbing moisture and exudates from wet wounds)
Absorption	Controlling the level of wound exudate and preventing tissue maceration by effectively absorbing excess fluid
Permeability	Facilitating gaseous exchange (water vapor, O_2_, CO_2_) to the wound bed to accelerate cellular activity
Protection	Preventing microbial infections that could impede the wound healing process and prolong its duration
Transparency	Enabling clinicians to visualize the wound and monitor the healing process
Mechanical robustness	Mimicking the structure of native skin while being rigid enough to allow fixation on the wound
Flexibility and elasticity	Adapting to body movements and minimizing patient discomfort and pain during application and dressing replacement
Adhesiveness	Providing good adhesion to healthy skin without sticking to the wound itself, facilitating easy removal after re-epithelialization, and preventing secondary injuries in the newly formed tissue
Biocompatibility	Minimizing the risk of immune reactions or side effects
Safety	Ensuring the absence of toxic substances that could cause damage or result in dire consequences; ensuring that products resulting from degradation are safe and follow the normal metabolic pathway
Cost-effectiveness	Supplying a cost-effective wound dressing solution
Availability	Widely available to all patients and healthcare centers

**Table 2:** Main sources for obtaining natural polymers and chemical synthesis pathways of synthetic polymers.

Polymers		Main Natural Sources/Chemical Synthesis Pathways	References
**Natural polymers**	**Collagen**	Bovine, porcine, red algae, fish, from species such as *Prionace glauca*, *Oreochromis niloticus*, and *Lophius litulon*, octopuses, starfish, jellyfish such as *Rhopilema esculentum*, polar bears, whales, seals, and marine sponges	[4,42]
	**Gelatin**	Bovine, porcine, caprine, mammalian tissues, squid, sponges, jellyfish, and snails	[17]
	**Silk Fibroin**	Cocoons of Mulberry silkworm *Bombyx mori* and the non-mulberry silkworm *Antheraea assama*	[43]
	**Alginate**	Brown marine algae such as *Laminaria*, *Ascophyllum*, and *Macrocystis*, red and green marine macroalgae, or bacteria like *Pseudomonas* and *Azotobacter*	[1,34,38,44]
	**Hyaluronic acid**	Human vitreous humor, joints in the umbilical cord, connective tissue	[45]
	**Bacterial cellulose**	Bacteria strains such as *Gluconacetobacter*, *Rhizobium*, *Agrobacterium*, *Sarcina*, and *Rhodobacter*	[1,16,46,47]
	**Dextran**	Bacteria, such as *Leuconostoc*, *Weissella*, *Lactobacillus*, and *Streptococcus*	[48,49]
	**Chitosan**	Insects, marine invertebrates, and crustaceans such as crabs, shrimp, and lobster, as well as in the cell walls of fungi	[9,16,50,51,52,53]
**Synthetic polymers**	**Polyurethane**	Result from the polyaddition polymerization between polyols (molecules containing hydroxyl groups) and isocyanates, in the presence of a chain extender and a catalyst	[54,55,56]
	**Polyethylene glycol**	Result from the condensation of ethylene oxide and water	[57,58]
	**Polyvinyl alcohol**	Results from the hydrolysis, aminolysis, or alcoholysis of vinyl acetate	[59]
	**Poly-ε-caprolactone**	Results from the ring-opening polymerization of ε-caprolactone	[60]
	**Poly-N-vinylpyrrolidone**	Results from high-pressure free-radical polymerizations	[61]
	**Poly(lactic-co-glycolic) acid**	Results from the blend of polylactic acid (derives from lactic acid and results from renewable sources such as corn, starch, or sugarcane) and polyglycolic acid (results from the hydrating carbonylation of formaldehyde, with H_2_SO_4_ as a catalyst, or from the pyrolysis of renewable sources such as sugar beets, sugarcane, cantaloupe, and grapes)	[9,54,60,62]

**Table 3:** Polymer critical analysis per critical attribute for wound healing.

Polymers		Critical Attributes												Eco-Friendly	References
		Moisture	Absorption	Permeability	Protection	Transparency	Mechanical Robustness	Flexibility and Elasticity	Adhesiveness	Biocompatibility	Safety	Cost-Effectiveness	Availability		
**Natural polymers**	Collagen	++	++	NA	+	+++	+	++	+	+++	++	+	++	√	[4,16,33,37,38,42,64,67,68,69,70,73,75,76,78,79]
	Gelatin	++	+++	NA	+	+++	+	++	+	+++	+++	++	++	√	[17,27,31,67,83,84,85,86,87,88,89,90,91,92,93,94,95,96,97,99]
	Silk Fibroin	++	++	++	+	CV	+++	+++	++	+++	+++	++	++	√	[16,17,54,57,60,102,103,104,105,106,107,108,109,110,112,113,114,115,116,117,119,120,121,122,123,124,125,126,127]
	Alginate	++	+++	++	+	+++	+	++	+	+++	+++	++	++	√	[1,16,21,34,38,39,44,53,128,129,130,131,132,133,134]
	Hyaluronic acid	++	+++	++	+	+++	+	++	+	+++	+++	+	++	√	[7,9,19,45,137,138,140,141,142,143,144,145,146,147]
	Bacterial cellulose	++	+++	++	+	NA	+++	++	NA	+++	+++	++	+++	√	[8,16,36,46,47,51,52,130,131,149,150,151,152,153,154,155]
	Dextran	++	+++	NA	+	+++	+	++	+	+++	+++	++	++	√	[130,133,158,159,160,161,162,167,168,169,170,171,172,173,174]
	Chitosan	++	++	++	++	NA	+	++	+++	+++	+++	++	+++	√	[9,11,16,38,46,50,51,52,53,67,78,98,130,131,132,133,134,137,154,176,177,178,179,180,181]
**Synthetic polymers**	Polyurethane	NA	NA *	++	+	+++	+++	+++	+	+++	+	+++	NA	X	[9,39,54,55,60,186,187,188,190,191,192,193,194,195,199,202]
	Polyethylene glycol	++	+++	++	+	+++	CV	++	+	+++	+++	++	NA	√	[2,17,19,30,41,54,57,58,60,209,210,211,212,213,214,215,216,223,224,226]
	Polyvinyl alcohol	++	+++	++	+	++	CV	+	CV	+++	+++	++	NA	√	[11,17,39,40,59,60,86,119,215,227,228,229,230,231,232,233,234,235,236,237,243,244]
	Poly-ε-caprolactone	NA	+	+	+	+	+++	++	+	++	+++	++	NA	√	[39,40,54,59,60,237,247,248,249,251,252,253,254]
	Poly-N-vinylpyrrolidone	NA	CV	+++	+	+++	CV	NA	++	++	+++	++	NA	√	[17,39,40,54,262,263,264]
	Poly(lactic-co-glycolic) acid	-	-	+	NA **	NA	++	NA	+	++	+++	NA	NA	√	[40,54,57,60,237,272,273,274]

**Key:** “-” means “does not have”; “+” means “weak”; “++” means “good”; “+++” means “excellent”; “√” means “applicable”; “X” means “not applicable”; CV—controversial; NA—not available. * There is only information about foams/sponges. ** There is only information about nanofibers.

## Data Availability

No new data were created or analyzed in this study. Data sharing is not applicable to this article.

## Abbreviations

**3D**	Three-dimensional
**BC**	Bacterial cellulose
**BCM**	Bacterial cellulose membrane
**ECM**	Extracellular matrix
**EGF**	Epidermal growth factor
**FGF**	Fibroblast growth factor
**GA**	Glycolic acid
**HA**	Hyaluronic acid
**NF-kB**	Nuclear Factor Kappa B
**PCL**	Poly-ε-caprolactone
**PEG**	Polyethylene glycol
**PGA**	Polyglycolic acid
**PLA**	Polylactic acid
**PLGA**	Poly(lactic-co-glycolic) acid
**PU**	Polyurethane
**PVA**	Polyvinyl alcohol
**PVP**	Poly-N-vinylpyrrolidone
**RGD**	Arginine–glycine–aspartic acid peptide sequences
**SF**	Silk fibroin
**TGF-β**	Transforming growth factor
**VEGF**	Vascular endothelial growth factor
